# Glycine–GLRA1–calmodulin signaling regulates endoplasmic reticulum calcium to sustain insulin secretion and β-cell function

**DOI:** 10.1093/lifemeta/loaf044

**Published:** 2025-12-19

**Authors:** Jiarui Zhang, Zehui Cao, Jinbao Yang, Kuo Zhu, Shuai Liu, Weijie Liu, Jin Lu, Hongfang Zhao, Wei Dai, Chunmei Chang, Haobin Ye, Xingrong Du, Qi Wang, Binghua Jiang, Sijia Wang, Li Jin, Tongjin Zhao, Peng Li, Antonio Vidal-Puig, Yuanting Zheng, Leming Shi, Linzhang Huang

**Affiliations:** State Key Laboratory of Genetics and Development of Complex Phenotypes, Shanghai Key Laboratory of Metabolic Remodeling and Health, Institute of Metabolism and Integrative Biology, Drug Clinical Trial Center, Shanghai Xuhui Central Hospital, Zhongshan-Xuhui Hospital, Fudan University, Shanghai 200438, China; State Key Laboratory of Genetics and Development of Complex Phenotypes, Shanghai Key Laboratory of Metabolic Remodeling and Health, Institute of Metabolism and Integrative Biology, Drug Clinical Trial Center, Shanghai Xuhui Central Hospital, Zhongshan-Xuhui Hospital, Fudan University, Shanghai 200438, China; State Key Laboratory of Genetics and Development of Complex Phenotypes, Shanghai Key Laboratory of Metabolic Remodeling and Health, Institute of Metabolism and Integrative Biology, Drug Clinical Trial Center, Shanghai Xuhui Central Hospital, Zhongshan-Xuhui Hospital, Fudan University, Shanghai 200438, China; State Key Laboratory of Genetics and Development of Complex Phenotypes, Shanghai Key Laboratory of Metabolic Remodeling and Health, Institute of Metabolism and Integrative Biology, Drug Clinical Trial Center, Shanghai Xuhui Central Hospital, Zhongshan-Xuhui Hospital, Fudan University, Shanghai 200438, China; State Key Laboratory of Genetics and Development of Complex Phenotypes, Shanghai Key Laboratory of Metabolic Remodeling and Health, Institute of Metabolism and Integrative Biology, Drug Clinical Trial Center, Shanghai Xuhui Central Hospital, Zhongshan-Xuhui Hospital, Fudan University, Shanghai 200438, China; State Key Laboratory of Genetics and Development of Complex Phenotypes, Shanghai Key Laboratory of Metabolic Remodeling and Health, Institute of Metabolism and Integrative Biology, Drug Clinical Trial Center, Shanghai Xuhui Central Hospital, Zhongshan-Xuhui Hospital, Fudan University, Shanghai 200438, China; Department of Endocrinology, The First Affiliated Hospital of Naval Medical University, Shanghai 200433, China; Department of Endocrinology, The First Affiliated Hospital of Naval Medical University, Shanghai 200433, China; State Key Laboratory of Genetics and Development of Complex Phenotypes, Shanghai Key Laboratory of Metabolic Remodeling and Health, Institute of Metabolism and Integrative Biology, Drug Clinical Trial Center, Shanghai Xuhui Central Hospital, Zhongshan-Xuhui Hospital, Fudan University, Shanghai 200438, China; State Key Laboratory of Genetics and Development of Complex Phenotypes, Shanghai Key Laboratory of Metabolic Remodeling and Health, Institute of Metabolism and Integrative Biology, Drug Clinical Trial Center, Shanghai Xuhui Central Hospital, Zhongshan-Xuhui Hospital, Fudan University, Shanghai 200438, China; State Key Laboratory of Genetics and Development of Complex Phenotypes, Shanghai Key Laboratory of Metabolic Remodeling and Health, Institute of Metabolism and Integrative Biology, Drug Clinical Trial Center, Shanghai Xuhui Central Hospital, Zhongshan-Xuhui Hospital, Fudan University, Shanghai 200438, China; State Key Laboratory of Genetics and Development of Complex Phenotypes, Shanghai Key Laboratory of Metabolic Remodeling and Health, Institute of Metabolism and Integrative Biology, Drug Clinical Trial Center, Shanghai Xuhui Central Hospital, Zhongshan-Xuhui Hospital, Fudan University, Shanghai 200438, China; State Key Laboratory of Genetics and Development of Complex Phenotypes, Shanghai Key Laboratory of Metabolic Remodeling and Health, Institute of Metabolism and Integrative Biology, Drug Clinical Trial Center, Shanghai Xuhui Central Hospital, Zhongshan-Xuhui Hospital, Fudan University, Shanghai 200438, China; Human Phenome Institute, Zhangjiang Fudan International Innovation Center, Metabonomics and Systems Biology Laboratory at Shanghai International Centre for Molecular Phenomics, Fudan University, Shanghai 200032, China; Academy of Medical Science, Tianjian Laboratory of Advanced Biomedical Sciences, School of Life Sciences, Zhengzhou University, Zhengzhou, Henan 450001, China; CAS Key Laboratory of Computational Biology, Shanghai Institute of Nutrition and Health, University of Chinese Academy of Sciences, Chinese Academy of Sciences, Shanghai 200031, China; State Key Laboratory of Genetics and Development of Complex Phenotypes, Shanghai Key Laboratory of Metabolic Remodeling and Health, Institute of Metabolism and Integrative Biology, Drug Clinical Trial Center, Shanghai Xuhui Central Hospital, Zhongshan-Xuhui Hospital, Fudan University, Shanghai 200438, China; State Key Laboratory of Genetics and Development of Complex Phenotypes, Shanghai Key Laboratory of Metabolic Remodeling and Health, Institute of Metabolism and Integrative Biology, Drug Clinical Trial Center, Shanghai Xuhui Central Hospital, Zhongshan-Xuhui Hospital, Fudan University, Shanghai 200438, China; Academy of Medical Science, Tianjian Laboratory of Advanced Biomedical Sciences, School of Life Sciences, Zhengzhou University, Zhengzhou, Henan 450001, China; Academy of Medical Science, Tianjian Laboratory of Advanced Biomedical Sciences, School of Life Sciences, Zhengzhou University, Zhengzhou, Henan 450001, China; Institute of Metabolic Science and MRC Metabolic Diseases Unit, University of Cambridge, Cambridge CB2 0QQ, United Kingdom; Cambridge University Nanjing Centre of Technology and Innovation, Nanjing, Jiangsu 210031, China; Centro de Investigacion Principe Felipe, Valencia 46012, Spain; State Key Laboratory of Genetics and Development of Complex Phenotypes, Shanghai Key Laboratory of Metabolic Remodeling and Health, Institute of Metabolism and Integrative Biology, Drug Clinical Trial Center, Shanghai Xuhui Central Hospital, Zhongshan-Xuhui Hospital, Fudan University, Shanghai 200438, China; State Key Laboratory of Genetics and Development of Complex Phenotypes, Shanghai Key Laboratory of Metabolic Remodeling and Health, Institute of Metabolism and Integrative Biology, Drug Clinical Trial Center, Shanghai Xuhui Central Hospital, Zhongshan-Xuhui Hospital, Fudan University, Shanghai 200438, China; International Human Phenome Institutes (Shanghai), Shanghai 200438, China; State Key Laboratory of Genetics and Development of Complex Phenotypes, Shanghai Key Laboratory of Metabolic Remodeling and Health, Institute of Metabolism and Integrative Biology, Drug Clinical Trial Center, Shanghai Xuhui Central Hospital, Zhongshan-Xuhui Hospital, Fudan University, Shanghai 200438, China

**Keywords:** glycine, GLRA1, insulin, calcium, T2D, glucose

## Abstract

Glycine, a non-essential amino acid, has been linked to improved metabolic health and enhanced insulin secretion, yet its mechanistic role in β-cell function remains poorly defined. Here, we identify a glycine–GLRA1–calmodulin signaling axis that regulates endoplasmic reticulum (ER) calcium homeostasis to support insulin biosynthesis and β-cell survival. Dietary glycine deficiency impairs insulin secretion, reduces islet mass, and worsens glucose intolerance, while overexpression of serine hydroxymethyltransferase 2 (*Shmt2*), a key glycine biosynthetic enzyme, increases circulating glycine, enhances insulin output, and improves glucose control. Conversely, β-cell-specific deletion of *Glra1* phenocopies glycine deficiency, disrupting ER calcium dynamics, amplifying ER stress, and impairing insulin gene expression and secretion. Mechanistically, GLRA1 interacts with calmodulin to sustain ER calcium levels and alleviate ER stress, preserving β-cell viability under metabolic stress. Human genetic and transcriptomic analyses reveal that *GLRA1* expression and variants are associated with insulin secretion and glycemic traits, underscoring clinical relevance. These findings establish glycine as a signaling metabolite that activates a receptor–calcium axis to maintain β-cell function, offering a mechanistic rationale for targeting GLRA1 or dietary glycine in diabetes therapy.

## Introduction

Glycine, a non-essential amino acid derived from serine, is increasingly recognized for its role in metabolic regulation [1]. Reduced circulating glycine levels are consistently associated with insulin resistance, type 2 diabetes (T2D), and obesity across diverse ­populations. As a precursor to critical metabolites—including gluta­thione, porphyrins, purines, heme, and creatine—glycine exhibits antioxidant, anti-inflammatory, cryoprotective, and immunomo­dulatory properties across various tissues [2]. Clinically, reduced circulating glycine levels are strongly associated with metabolic syndrome, including T2D, impaired glucose tolerance, insulin resistance, obesity, and overweight, across diverse populations [3–8]. Moreover, interventions targeting these conditions—whether dietary or pharmacological—frequently elevate glycine levels, highlighting its therapeutic potential [9–11]. Notably, glycine supple­mentation enhances insulin secretion [12, 13], suggesting a direct role in glucose homeostasis. However, the molecular mechanism linking glycine to insulin production remains incompletely understood.

Glycine exerts many of its effects via glycine receptors (GlyRs), a family of pentameric ligand-gated ion channels classically associated with inhibitory neurotransmission in the central nervous system (CNS) [14]. These receptors are typically composed of four α subunits and one β subunit. While primarily studied in neurons, GlyRs are also expressed in peripheral tissues, including the pancreas [2]. In human pancreatic islets, the GlyR α1 subunit GLRA1 is selectively enriched in β-cells [15–17]. Though glycine is known to activate GlyRs, resulting in Cl^−^ influx and membrane hyperpolari­zation in neurons, its role in non-neuronal cells, particularly in relation to insulin regulation, is less well understood. Significantly, GLRA1 expression in isolated human islets correlates with insulin production [18], yet whether GLRA1 contributes to insulin biosynthesis or secretion *in vivo* has not been determined.

In β-cells, calcium (Ca^2+^) signaling is a key determinant of insulin secretion. The endoplasmic reticulum (ER) not only serves as the site of proinsulin synthesis but also acts as the primary intracellular Ca^2+^ reservoir. Adequate ER Ca^2+^ levels are critical for insulin exocytosis and for maintaining ER homeostasis [16, 19]. Conversely, ER Ca^2+^ depletion induces ER stress, a major contributor to β-cell dysfunction and T2D pathogenesis [20–22]. Although glycine has been implicated in the regulation of ER stress via calcium signaling [23], the precise contribution of GLRA1 to ER calcium dynamics and insulin production remains unknown.

Here, we identify glycine as a signaling metabolite that enhances insulin biosynthesis and secretion through a GLRA1–calmodulin–ER calcium axis. We show that GLRA1 activation increases ER calcium content, alleviates ER stress, and preserves β-cell mass and function, ultimately improving glucose homeostasis.

## Results

### Circulating glycine levels are inversely associated with hyperglycemia

**Figure 1:**
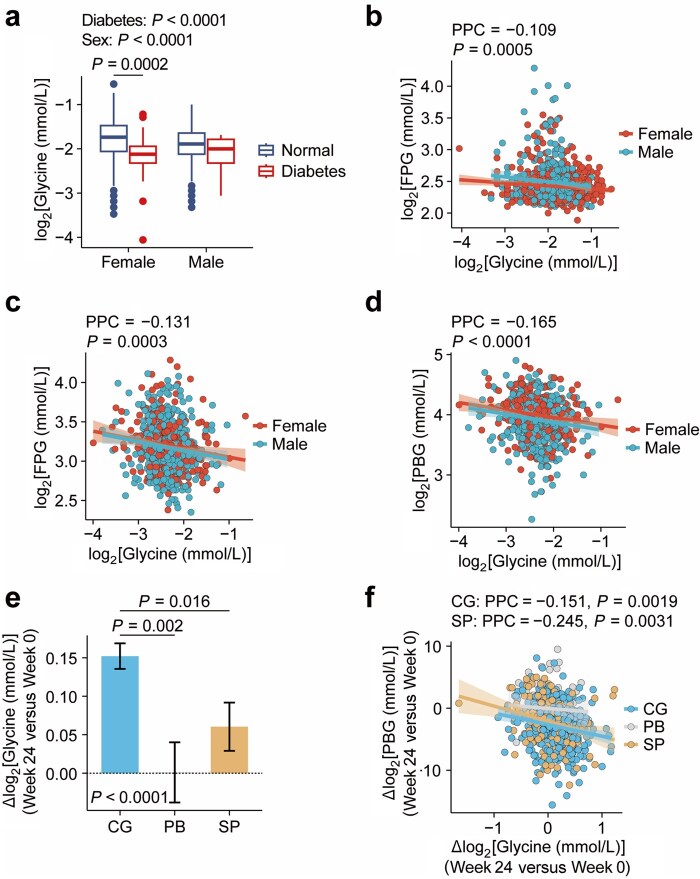
Circulating glycine levels are inversely associated with hyperglycemia. (a) Plasma glycine in non-diabetic (*n *= 38) and diabetic (*n *= 982) individuals in the NSPT cohort. The notch represents a confidence interval around the median as the median ± 1.58 × inter quartile range (IQR)/sqrt (*n*). (b) Partial Pearson’s correlation (PPC) between log-transformed fasting plasma glucose (FPG, mmol/L) and log-transformed plasma glycine (mmol/L) in the NSPT cohort, adjusted for sex and age. Red: female (*n *= 617); blue: male (*n *= 389). (c and d) PPC between log-transformed FPG (c) or PBG (d) and log-transformed plasma glycine (mmol/L) in the ChiHOPE cohort, adjusted for sex and age. Red: female (*n *= 265); blue: male (*n *= 469). (e) Changes in plasma glycine levels after chiglitazar sodium (*n *= 418), placebo (*n *= 81), and sitagliptin (100 mg/day, *n *= 146) treatment for 24 weeks, adjusted for baseline glycine levels, age, sex, and clinical trial. (f) PCC between log-transformed changes in PBG and glycine levels (mmol/L) in people receiving chiglitazar sodium (*n *= 418), placebo (*n *= 81), and sitagliptin (100 mg/day, *n *= 146) treatment for 24 weeks, adjusted for sex and age. Values shown are log_2_(concentration in mmol/L). Data are expressed as mean ± SEM.

To investigate the relationship between circulating glycine and glucose metabolism, we first analyzed data from the National Survey of Physical Traits (NSPT) cohort (*n *= 1020; 394 males, 626 females) [24]. Plasma glycine levels were significantly lower in individuals with T2D compared to normoglycemic controls, even after adjusting for age and sex (Fig. 1a). Glycine levels were inversely correlated with fasting plasma glucose (FPG) concentrations (Fig. 1b), suggesting a potential role for glycine in glucose regulation.

We next examined an independent cohort from the Chiglitazar perturbed Human multi-Omics ProfilE (ChiHOPE; fudan-pgx.org/chihope/), which includes multi-omic profiling of individuals with T2D. Consistent with NSPT findings, glycine levels were negatively associated with both FPG and 2-h postprandial blood glucose (PBG) in both males and females (Fig. 1c and d). Notably, treatment with the glucose-lowering drug Chiglitazar (48 mg/day), a pan-peroxisome proliferator-activated receptor (pan-PPAR) agonist, significantly increased plasma glycine levels compared to placebo (Fig. 1e), sitagliptin served as a positive control for glucose lowering interventions. After 24 weeks, reductions in PBG strongly paralleled increases in glycine levels (Fig. 1f), suggesting a link between glycine and improved postprandial glucose handling. Since PBG is a surrogate for postprandial insulin secretion and β-cell function, these data raise the possibility that elevated glycine levels may contribute to enhanced islet function and insulin release *in vivo*.

### Glycine deficiency impairs insulin secretion and exacerbates glucose intolerance

**Figure 2:**
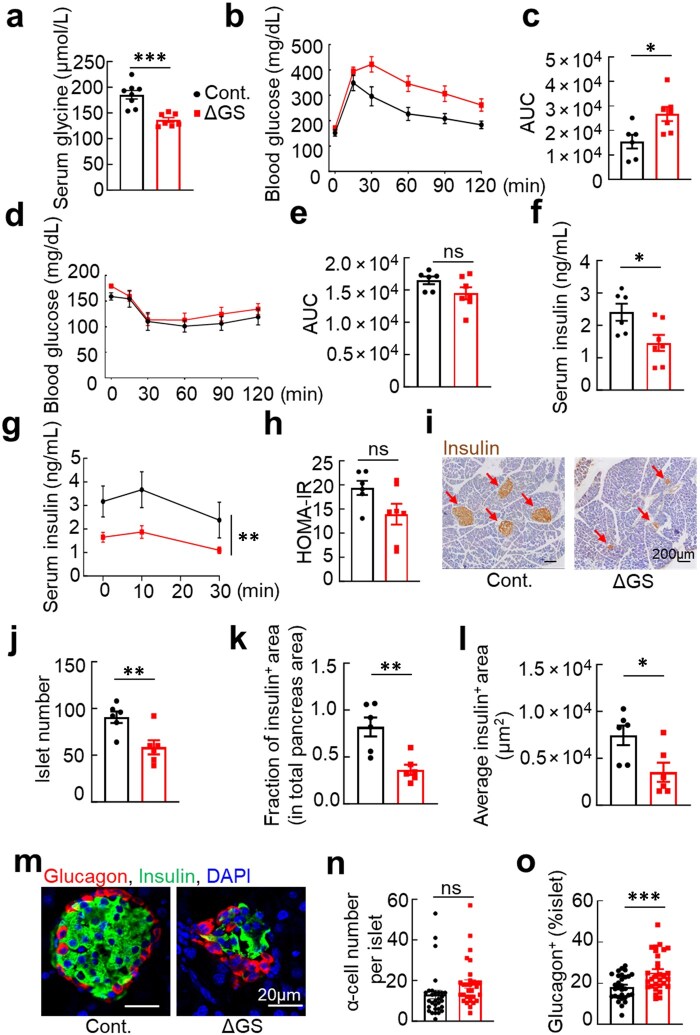
Glycine deficiency impairs insulin secretion and exacerbates glucose intolerance. (a) Serum glycine levels in mice fed control versus glycine-deficient diet. *n *= 8 and 7, respectively. (b and c) GTT (b) and its AUC quantification (c). *n *= 6 and 7, respectively. (d and e) ITT and its AUC quantification. *n *=  6 and 7, respectively. (f) Fasting blood insulin levels. *n *= 6 and 7, respectively. (g) GSIS. *n *= 6 for both groups. (h) HOMA-IR in mice fed control versus glycine-deficient diet, *n *= 6 and 7, respectively. (i–l) Pancreas insulin IHC staining (i), quantification of islet number per section (j), fraction of insulin-positive area relative to total pancreatic area (k), and the average insulin-positive area per islet (l). *n *= 6 for both groups. Red arrows indicate islets. Scale bar: 200 μm. Islet numbers and area were counted from at least 10 sections. (m–o) Immunofluorescence images of insulin and glucagon staining (m), α-cell number per islet (n), and the quantification of glucagon-positive area (o). *n *= 30 islets from five mice per group. Scale bar: 20 μm. Male mice of 4–5 weeks old were placed on control or ΔGS HFD for 12 weeks, followed by harvesting blood for glycine determination or fasting for 4–6 h for GTT, ITT, or GSIS assays. For GTT, 1 g/kg dextrose was employed, while for ITT, 1 U/kg insulin was injected into mice. Blood glucose was determined using a glucose meter at the indicated time points. Data are expressed as mean ± SEM. *P < 0.05; ** *P *< 0.01; *** *P *< 0.001; ns, not significant. See also Supplementary Figs. S1–S4.

Circulating glycine originates from dietary intake, endogenous biosynthesis, and catabolism [25]. Given its inverse association with hyperglycemia in humans, we hypothesized that reduced dietary glycine could impair glucose homeostasis and insulin secretion. To test this, we fed wild-type (WT) mice a high-fat diet (HFD) lacking both glycine and serine (ΔGS), creating a state of dietary glycine deficiency. Mice on the ΔGS diet exhibited significantly reduced serum glycine levels compared to HFD-fed control mice (Fig. 2a). Importantly, glycine-deficient mice displayed pronounced glucose intole­rance, as shown by glucose tolerance tests (GTTs) (Fig. 2b and c), phenocopying features of human T2D. To determine whether elevated glucose levels arose from increased hepatic gluconeogenesis, we performed ­pyruvate tolerance tests (PTTs). Blood glucose responses following pyruvate challenge were comparable between ΔGS-fed and control mice (Supplementary Fig. S1a and b), indicating that glycine ­deficiency did not enhance hepatic glucose output. Consistently, hepatic expression of key gluconeogenic genes remained unchanged. Histological analysis of liver sections also revealed no overt morphological differences between groups (Supplementary Fig. S1c and d), supporting the notion that glycine deficiency does not directly affect hepatic metabolism. Together, these findings suggest that dietary glycine deficiency worsens glucose homeostasis through extrahepatic mechanisms, likely involving impaired insulin secretion.

To determine whether the glucose intolerance observed in glycine-deficient mice was due to altered insulin sensitivity, we performed insulin tolerance tests (ITTs). Blood glucose clearance following insulin administration was comparable between mice on the standard and ΔGS diets (Fig. 2d and e), indicating that glycine deficiency does not affect systemic insulin sensitivity. In contrast, glycine-deficient mice exhibited significantly reduced fasting insulin levels and impaired glucose-stimulated insulin secretion (GSIS) re­lative to controls (Fig. 2f and g), while the homeostasis model assessment of insulin resistance (HOMA-IR) remained unchanged (Fig. 2h), suggesting that dietary glycine is essential for proper insulin production and secretory function. Histological analysis further revealed that glycine deprivation led to a decrease in both pancreatic islet number and total islet mass, accompanied by an increased α-cell to β-cell ratio (Fig. 2i–o). These findings indicate that glycine also influences islet morphology and cellular composition, in addition to its role in β-cell function. Importantly, glycine deficiency did not affect overall metabolic health: body composition, plasma cholesterol and triglyceride (TG) levels, adipocyte size, and whole-body energy expenditure (EE) were all unchanged (Supplementary Fig. S1e–m). Together, these results identify glycine as a key regulator of ­pancreatic islet function and insulin secretion, acting independently of ­peripheral insulin sensitivity or systemic lipid metabolism.

To further investigate the role of glycine in glucose regulation, we next examined the effects of elevated systemic glycine levels by enhancing its endogenous biosynthesis. Given that serine is converted to glycine via the mitochondrial enzyme serine hydroxymethyltransferase 2 (SHMT2) [26], we delivered an adeno-associated virus (AAV) encoding *Shmt2* cDNA to WT mice. Ectopic *Shmt2* expression significantly increased circulating glycine levels ­(Supplementary Fig. S2a and b). Under standard chow conditions, *Shmt2*-overexpressing mice exhibited no significant change in glucose tolerance. However, under HFD stress, these mice showed markedly improved glucose regulation despite increased body weight due to elevated fat mass (Supplementary Fig. S2c–f). Notably, adipocyte size remained unchanged, consistent with earlier observations that glycine does not alter adipocyte morphology. Hepatic lipid accumulation and gluconeogenic activity were unaffected, and ITT or PTT revealed no differences (Supplementary Fig. S2g–r). These findings suggest that the glycemic improvements in *Shmt2*-overexpressing mice are not due to enhanced insulin sensitivity or reduced hepatic glucose production. In contrast, fasting insulin levels were significantly elevated in *Shmt2*-overexpressing mice ­without alteration in HOMA-IR, and GSIS was markedly enhanced (Supplementary Fig. S3a–c), indicating increased β-cell output. ­Histological analysis confirmed an increase in islet number and total islet mass (Supplementary Fig. S3d–j), providing a structural basis for the elevated insulin production. Together, these data demonstrate that genetic enhancement of ­glycine biosynthesis improves glucose tolerance predominantly by promoting insulin secretion that is ­independent of peripheral insulin sensitivity or hepatic metabolism.

To determine whether glycine acts in an autocrine manner within β-cells [18] to regulate insulin production, we selectively overexpressed *Shmt2* in pancreatic β-cells. We used *Ins2*-Cre mice injected with an AAV encoding a Cre-dependent Flip-Excision (FLEx) *Shmt2* vector (Supplementary Fig. S4a). Isolated islets from these mice exhibited robust SHMT2 overexpression, while hepatic SHMT2 levels remained unchanged, confirming β-cell specificity (Supplementary Fig. S4b and c). Although circulating glycine concentrations were unaffected, β-cell-specific *Shmt2* overexpression significantly improved glucose tolerance compared to control mice (Supplementary Fig. S4d–f), without altering insulin sensitivity (Supplementary Fig. S4g–i). Food intake, body weight, and body composition also remained unchanged (Supplementary Fig. S4j–l), indicating that the metabolic improvement was not due to systemic effects. Importantly, β-cell *Shmt2* overexpression led to increased insulin production and enhanced islet architecture, including higher islet number, increased β-cell ratio, and larger average islet size (Supplementary Fig. S4m–r). These findings demonstrate that localized glycine biosynthesis within β-cells is sufficient to stimulate insulin secretion and improve glucose homeostasis, independent of changes in peripheral metabolism.

### β-cell GLRA1 is required for insulin secretion and glucose homeostasis

Glycine binds to GlyRs localized on the plasma membrane. To determine how glycine signals within pancreatic β-cells, we investigated the expression of GlyR subunits, which form pentameric chloride channels on the plasma membrane. Using the rat β-cell line INS1, we assessed the expression of the four α subunit isoforms (*Glra1*−*4*) and the β subunit (*Glrb*). We detected transcripts for *Glra1*, *Glra3*, and *Glrb*, whereas *Glra2* and *Glra4* were undetectable ­(Supplementary Fig. S5a). These expression patterns are consistent with previously reported GlyR profiles in human β-cells [17, 27, 28]. Because GLRB (the β subunit) is ubiquitously co-expressed with functional α subunits, we focused our investigation on *Glra1* ­(encoding the α1 subunit), which was the most abundantly expressed. We hypothesized that GLRA1 mediates the β-cell response to glycine and contributes to insulin secretion and glucose regulation.

**Figure 3:**
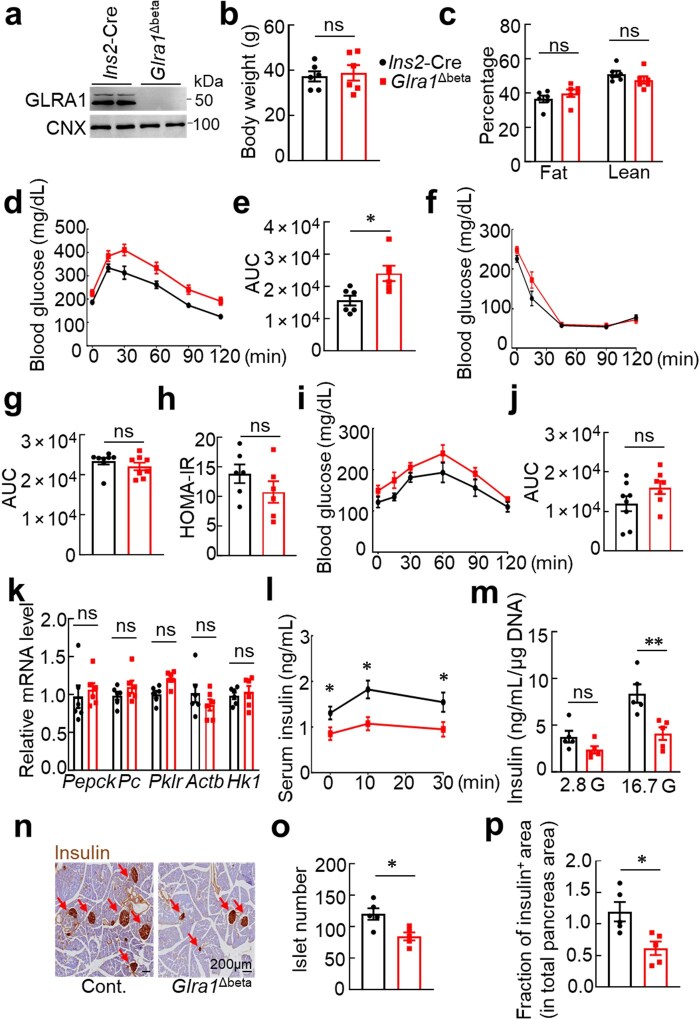
β-cell GLRA1 is required for insulin secretion and glucose homeostasis. (a) Representative immunoblotting image of GLRA1 in islets. The ­experiment was independently repeated for two times. (b and c) Body mass (b) and the percentage of fat and lean mass (c). *n *= 6 for both groups. (d and e) GTT (d) and its AUC quantification (e). *n *= 6 for both groups. (f and g) ITT (f) and its AUC quantification (g). *n *= 8 for both groups. (h) HOMA-IR in mice expressing or lacking *Glra1* in β-cells. *n *= 6 for both groups. (i and j) PTT (i) and its AUC quantification (j). *n *= 8 and 7, respectively. (k) Hepatic gluconeogenesis gene expression. *n *= 6 for both groups. (l) *In vivo* GSIS of mice expressing or lacking *Glra1* in β-cells. *n *= 6 for both groups. (m) GSIS in isolated islets from mice expressing or lacking *Glra1* in β-cells. *n *= 5 for both groups. (n−p) Pancreas insulin IHC staining (n), quantification of islet number per section (o), and fraction of insulin-positive area relative to total pancreatic area (p). *n *= 5 for both groups. Red arrows indicate islets. Scale bar: 200 μm. Islet numbers and areas were counted from at least 10 sections. Male mice of 4–5 weeks old expressing or lacking *Glra1* in β-cells were placed on HFD for 12 weeks, followed by GTT, ITT, or GSIS assays. For GTT, 1 g/kg dextrose was employed, while for ITT, 1 U/kg insulin was injected into mice. Blood glucose was determined using a glucose meter at the indicated time points. Data are expressed as mean ± SEM. *P < 0.05; **P < 0.01; ***P < 0.001; ns, not significant. See also ­Supplementary Figs. S5–S7.

To investigate the role of GLRA1 in β-cell-mediated glucose regula­tion, we generated β-cell-specific *Glra1* knockout mice (*Glra1*^Δbeta)^. GLRA1 expression was markedly reduced in isolated islets from *Glra1*^Δbeta^ mice, confirming efficient deletion (Fig. 3a). Under chow diet-fed conditions, *Glra1*^Δbeta^ mice exhibited comparable GTT and ITT to *Ins2*-Cre control mice (Supplementary Fig. S5b and c). However, after 12 weeks of HFD feeding, mice lacking GLRA1 in β-cells exhibited significantly impaired glucose tolerance relative to control mice (Fig. 3b–e). ITT, HOMA-IR, and PTT analyses revealed no alterations in peripheral insulin sensitivity or hepatic gluconeogenesis (Fig. 3f–k), suggesting that the glucose intolerance is not driven by systemic metabolic changes. Strikingly, GSIS was severely impaired in *Glra1*^Δbeta^ mice. In isolated islets, high-glucose stimulation elicited robust insulin release in control mice but failed to do so in GLRA1-deficient islets (Fig. 3l and m), demonstrating that GLRA1 is essential for coupling glucose sensing to insulin exocytosis. Histological analysis revealed that *Glra1*^Δbeta^ mice exhibited reduced islet number and total islet mass, along with an increased proportion of glucagon-positive α-cells and serum glucagon levels (Fig. 3n–p; Supplementary Fig. S5b–h), indicating that GLRA1 is also important for maintaining islet architecture and β-cell identity. Together, these findings establish GLRA1 as a critical mediator of glycine signaling in β-cells, required for insulin production, islet integrity, and systemic glucose homeostasis.

To confirm the role of glycine–GLRA1 signaling in insulin secretion, we performed GSIS assays in INS1-cells. In the absence of glycine, insulin release in response to high glucose was significantly reduced (Supplementary Fig. S5i). In contrast, glycine supplementation markedly enhanced insulin secretion under high-glucose conditions (Supplementary Fig. S5j). To determine whether this effect was mediated by GLRA1, we silenced *Glra1* in INS-1 cells. Knockdown of *Glra1* had no impact on basal GSIS but completely abolished the glycine-induced enhancement of insulin release (Supplementary Fig. S5k). Similarly, pharmacological inhibition of GLRA1 with strychnine, a competitive GlyR antagonist, prevented the ability of glycine to augment insulin secretion (Supplementary Fig. S5l). Of note, glycine deficiency/*Glra1* knockdown leads to a significant reduction in the number of docked insulin granules at the plasma membrane, without significantly altering the total gra­nule number (Supplementary Fig. S6a–k). Collectively, these results establish that glycine potentiates insulin secretion through activation of GLRA1, confirming a direct role for this receptor in β-cell glycine sensing and insulin regulation.

### Glycine–GLRA1 signaling alleviates ER stress to promote insulin biosynthesis

**Figure 4:**
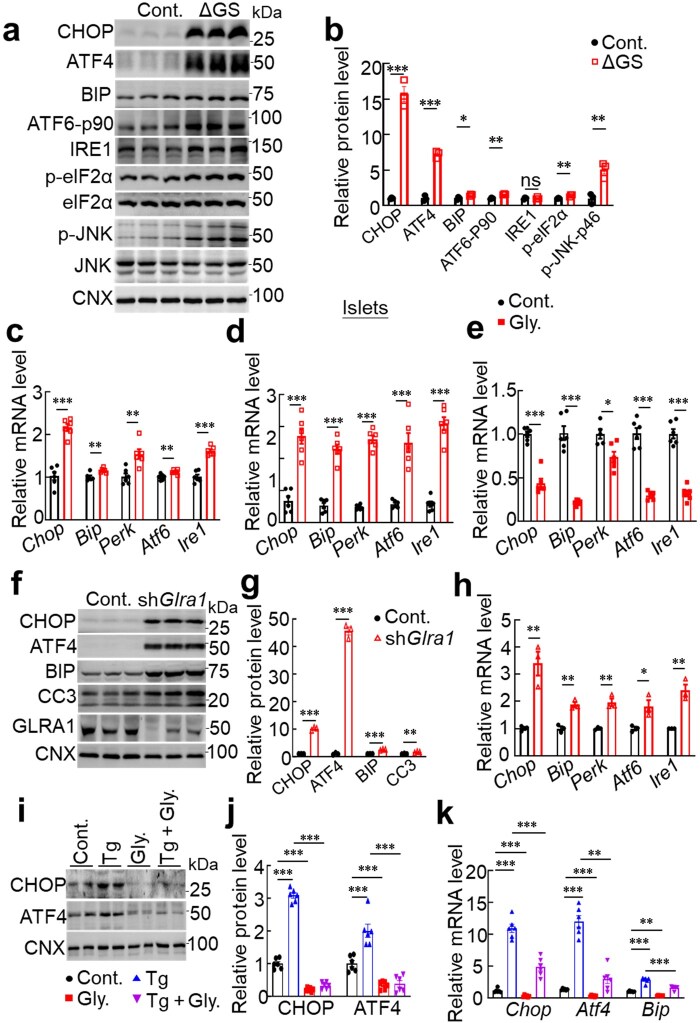
Glycine−GLRA1 signaling alleviates ER stress to promote insulin biosynthesis. (a and b) Representative immunoblotting of ER stress markers (a) and its quantification (b) following the treatment of glycine/serine deficiency for 16 h. GSD: glycine and serine deficiency. *n *= 3 for both groups. (c and d) Relative mRNA levels of ER stress markers in the INS1 cells (c) or isolated islets (d) from HFD-fed mice cultured in glycine-deficient medium. *n *= 6 for both groups. (e) Relative mRNA levels of ER stress markers in the presence or absence of glycine supplementation. *n *= 6 for both groups. (f and g) Representative immunoblotting of ER stress markers (f) and its quantification (g) in cells expressing or lacking *Glra1*. The experiment was independently repeated for three times. (h) The mRNA levels of ER stress markers in cells expressing or lacking *Glra1*. *n *= 3 for both groups. (i–k) Immunoblotting of ER stress markers (i), quantification (j), and the mRNA levels (k) in the presence or absence of Tg stimulation (1 μmol/L) for 1 h, following 10 mmol/L glycine treatment overnight in INS1 cells. *n *= 6 for all the groups. Data are expressed as mean ± SEM. *P < 0.05; **P < 0.01, ***P < 0.001; ns, not significant. See also Supplementary Fig. S8.

Having established that glycine and GLRA1 are essential for insulin secretion and glucose homeostasis, we next investigated the underlying mechanism by which glycine regulates insulin production. Given that ER stress is a well-established suppressor of insulin biosynthesis [20] and inversely correlates with insulin production, we examined whether glycine modulates ER stress responses in β-cells. Both INS1 cells and islets subjected to glycine/serine deprivation exhibited significant upregulation of classical ER stress markers, including CCAAT/enhancer-binding protein (C/EBP) homologous protein (CHOP), immunoglobulin heavy chain binding protein (BIP, also known as 78-kDa glucose-regulated protein (GRP78)), and activating transcription factor 6 (ATF6) (Fig. 4a–d). Conversely, glycine supplementation reduced the expression of these markers, suggesting that glycine mitigates ER stress (Fig. 4e). Similarly, *Shmt2* overexpression attenuated ER stress in islets from HFD-fed mice (Supplementary Fig. S7a–d). Consistent with these observations, glycine deprivation suppressed insulin mRNA expression, whereas glycine supplementation enhanced insulin transcript levels (Supplementary Fig. S7e and f), linking ER stress resolution to restored insulin biosynthesis. Together, these findings indicate that glycine alleviates ER stress to support insulin gene transcription, thereby preserving β-cell function and contributing to improved glucose homeostasis.

To determine whether GLRA1 mediates the effect of glycine on ER stress, we assessed stress markers in β-cell-specific *Glra1* knockout mice. Deletion of *Glra1* significantly elevated the expression of key ER stress proteins, including CHOP, BIP, ATF4, and cleaved caspase-3 (CC3), along with an increased transcription of ER stress-associated genes and the staining of CHOP and BIP in pancreas sections (Fig. 4f–h; Supplementary Fig. S7g and h). Notably, *Glra1* knockout in islets fully abolished glycine-attenuated ER stress (Supplementary Fig. S7i). These findings indicate that GLRA1 is required to maintain ER homeostasis in β-cells. Consistently, pharmacological inhibition of GLRA1 using strychnine abolished glycine-induced enhancement of insulin gene expression, as shown by reduced *Ins1* and *Ins2* mRNA levels (Supplementary Fig. S7j). Together, these data demonstrate that GLRA1 activation is essential for glycine-mediated alleviation of ER stress, which in turn supports insulin biosynthesis and β-cell function.

Glycine uptake into mammalian cells is mediated primarily by glycine transporters 1 and 2 (GlyT1/SLC6A9 and GlyT2/SLC6A5, respectively), with GlyT1 being the predominant isoform expressed in pancreatic β-cells. To determine whether intracellular glycine transport contributes to its insulinotropic effects, we selectively knocked down *Glyt1* in β-cells. In contrast to *Glra1* silencing, *Glyt1* knockdown left ER stress marker proteins unaltered yet triggered subtle transcriptional upregulation of the unfolded protein response (UPR) genes *Chop* and PKR-like ER kinase (*Perk*) (Supplementary Fig. S8a–e). Moreover, deletion of GlyT1 did not impair the ability of exogenous glycine to stimulate insulin secretion (Supplementary Fig. S8f). These results indicate that GlyT1-mediated glycine transport is dispensable for the action of glycine on insulin production and highlight that the effect is driven primarily by receptor-mediated signaling via GLRA1.

To investigate whether glycine mitigates ER stress by preserving ER calcium homeostasis, we treated β-cells with thapsigargin (Tg), a sarco/endoplasmic reticulum Ca^2+^-ATPase (SERCA) inhibitor that blocks ER calcium reuptake and triggers ER stress [29]. As expected, Tg treatment robustly upregulated the ER stress markers, including CHOP, ATF4, and BIP. In contrast, glycine treatment alone reduced their expression, and co-treatment with glycine significantly attenuated Tg-induced CHOP, ATF4, and BIP upregulation (Fig. 4i–k; ­Supplementary Fig. S9a–c), indicating that glycine can counteract pharmacologically induced ER stress. Given the mechanism of action of Tg, these findings suggest that glycine alleviates ER stress, at least in part, by restoring or maintaining ER calcium levels under stress conditions.

### GLRA1 activation enhances ER calcium levels via calmodulin

**Figure 5:**
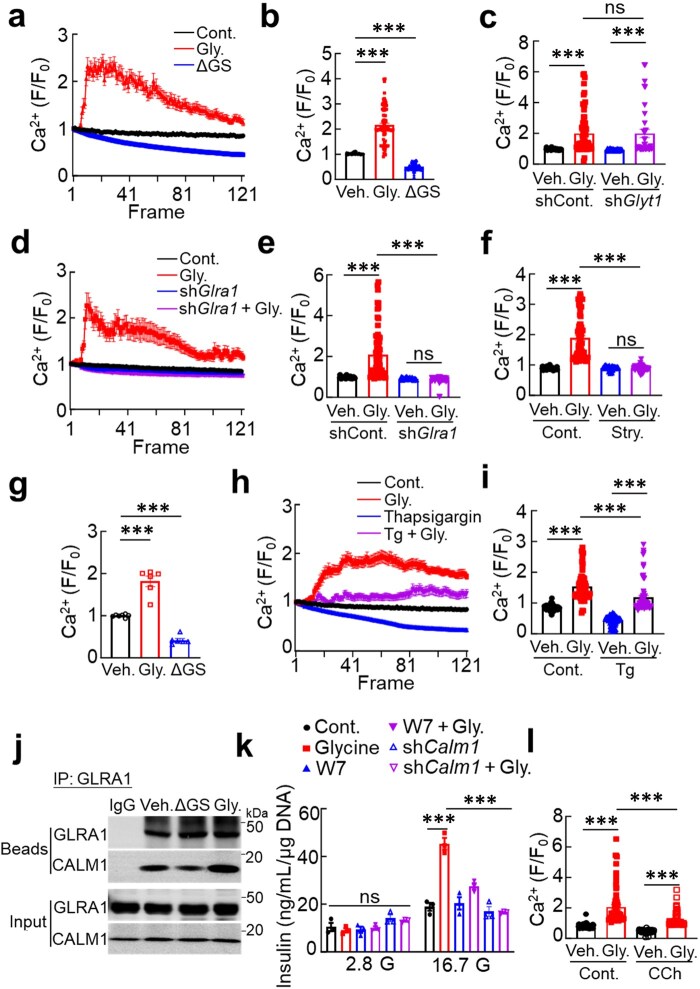
GLRA1 activation enhances ER calcium levels via calmodulin. (a and b) ER calcium (a) and its quantification (b) in INS1 cells treated with glycine-proficient (1 mmol/L) or glycine-deficient medium. *n *= 58, 88, and 133 cells, respectively. (c) Quantification of ER calcium in cells expressing or lacking *Glyt1* in the presence or absence of glycine (1 mmol/L). *n *= 50, 70, 80, and 37 cells, respectively. (d and e) ER calcium (d) and its quantification (e) in cells expressing or lacking *Glra1* in the presence or absence of glycine (1 mmol/L). *n *= 40, 73, 49, and 72 cells, respectively. (f) Quantification of ER calcium in cells treated with or without GLRA1 antagonist, strychnine (3 μmol/L). *n *= 57, 72, 53, and 61 cells, respectively. (g) Quantification of ER calcium in islets isolated from HFD-fed mice, treating with glycine-proficient (1 mmol/L) or glycine-deficient medium, 10 islets per well. *n *= 7, 6, and 6, respectively. (h and i) ER calcium (h) and its quantification (i) in response to Tg (1 μmol/L) with or without glycine (1 mmol/L) treatment. *n *= 61, 69, 62, and 70 cells, respectively. (j) GLRA1 and calmodulin interaction in response to glycine stimulation (0, 0.133, and 5 mmol/L). The experiment was independently repeated for three times. (k) GSIS in cells treated with calmodulin inhibitor W7 (50 μmol/L) or shRNA targeted against calmodulin. *n *= 3 for all the groups. (l) Quantification of ER calcium in cells treated with CCh (1 mmol/L) in the presence or absence of glycine. *n *= 66, 68, 80, and 65, respectively. ER calcium was monitored using CEPIA1er in INS1 cells, while Mag-Fluo-4 staining was employed in islets. Data are expressed as mean ± SEM. *P < 0.05; **P < 0.01; ***P < 0.001; ns, not significant. See also Supplementary Fig. S9.

Previous studies have shown that GLRA1 activation elevates intracellular calcium levels [18]. Given that the ER serves as the major intracellular calcium reservoir [30], we investigated whether glycine influences ER calcium dynamics. To this end, we used calcium-measuring organelle-entrapped protein indicator 1 in the ER (CEPIA1er) [31, 32], a genetically encoded ER calcium sensor, to monitor calcium flux in real time. Glycine supplementation significantly increased ER calcium signals, whereas glycine deprivation reduced ER calcium flux (Fig. 5a and b), indicating that glycine plays a key role in maintaining ER calcium homeostasis. These findings suggest that GLRA1 activation sustains ER calcium levels, likely contributing to its role in alleviating ER stress and supporting insulin biosynthesis.

To directly compare the roles of GLRA1 and GlyT1 in ER calcium regulation, we examined their respective contributions to glycine-induced calcium dynamics. Knockdown of *Glyt1* did not affect ER calcium levels, and glycine supplementation enhanced ER calcium signaling irrespective of *Glyt1* expression (Fig. 5c), indicating that glycine transport is not required for its calcium-regulating effects. In contrast, deletion of *Glra1* or pharmacological inhibition of GLRA1 using strychnine completely abolished glycine-induced ER calcium elevation (Fig. 5d–f). Notably, glycine-enhanced calcium via GLRA1 was also evident in isolated islets stained with Mag-Fluo-4 (Fig. 5g; Supplementary Fig. S9d). Consistently, glycine increased cytosolic calcium in a GLRA1-dependent pathway. However, ATP was dispensable for this process (Supplementary Fig. S9e and f). These findings demonstrate that GLRA1 is essential for mediating the effects of extracellular glycine on ER calcium homeostasis.

As expected, Tg, a SERCA inhibitor, significantly reduced ER calcium levels compared to untreated control group, confirming its role in blocking ER calcium uptake. Given the ability of glycine to alleviate ER stress, we next tested whether it could counteract Tg-induced calcium depletion. Co-treatment with glycine restored ER calcium dynamics in Tg-treated cells and reversed the associated upregulation of ER stress markers (Fig. 5h and i). These results indicate that glycine mitigates ER stress by rescuing ER calcium homeostasis, further supporting its role as a calcium-regulating signaling metabolite in β-cells.

Calmodulin is a key regulator of ER calcium homeostasis [33]. To investigate whether GLRA1 influences ER calcium through calmodulin, we examined their interaction under varying glycine conditions. Glycine treatment enhanced the physical association between GLRA1 and calmodulin, whereas glycine deprivation weakened this interaction (Fig. 5j), suggesting that glycine facilitates GLRA1–calmodulin coupling as part of its signaling mechanism. Importantly, silencing or pharmacological inhibition of calmodulin abolished glycine-induced ER calcium elevation and significantly impaired glycine-mediated enhancement of GSIS (Fig. 5k; Supplementary Fig. S9g). Calmodulin directly binds to and inhibits inositol 1,4,5-trisphosphate (IP_3_) receptor (IP_3_R) channels [34, 35], thereby reducing ER Ca^2+^ efflux to maintain or increase ER calcium stores. We then determined whether IP_3_R activity is required for glycine-mediated ER Ca^2+^ retention and ER stress attenuation. Indeed, pharmacological promotion of IP_3_R-mediated Ca^2+^ release using carbamylcholine (CCh) substantially abolished the protective effects of glycine on ER calcium and ER stress (Fig. 5l; Supplementary Fig. S9h). These findings establish that glycine modulates ER stress via GLRA1–calmodulin–IP_3_R axis to support insulin biosynthesis and secretion in pancreatic β-cells.

### GLRA1 signaling preserves β-cell mass by promoting survival and proliferation

**Figure 6:**
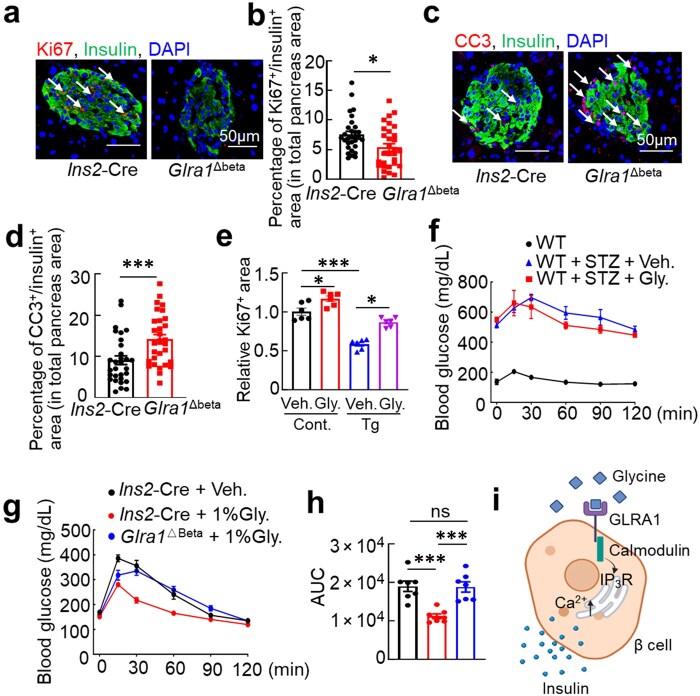
GLRA1 signaling preserves β-cell mass by promoting survival and proliferation. (a and b) Representative immunofluorescence images (a) and its quantification (b) of Ki67 staining for islets expressing or lacking *Glra1* in the pancreas. *n *= 30 for both groups. (c and d) Representative immunofluorescence images (c) and its quantification (d) of CC3 staining for islets expressing or lacking *Glra1* in the pancreas. *n *= 30 for both groups. (e) Relative Ki67 staining for INS1 cells treated with vehicle or glycine in the presence or absence of Tg (1 μmol/L). *n *= 6 for all groups. (f) GTT in mice treated with glycine (1%, w/v) following STZ administration. *n *= 6, 5, and 6, respectively. (g and h) GTT (g) and its AUC quantification (h) in mice expressing or lacking *Glra1* in islets placed on 1% glycine drink water. *n *= 7 for all groups. (i) A diagram illustrating that glycine–GLRA1 signaling drive calmodulin-dependent ER calcium elevation, which facilitates insulin biosynthesis and secretion. Data are expressed as mean ± SEM. *P < 0.05; **P < 0.01; ***P < 0.001; ns, not significant. See also Supplementary Fig. S10.

Consistent with the role of glycine in enhancing pancreatic islet number and β-cell mass, β-cell-specific deletion of *Glra1* led to a marked reduction in both parameters. GLRA1 deficiency exacerbated ER stress, disrupted ER calcium dynamics, and increased β-cell death—all key contributors to diabetes pathogenesis. In T2D, chronic ER stress is a major driver of β-cell apoptosis [36]. To directly assess the role of GLRA1 in β-cell survival, we analyzed proliferation and apoptosis in *Glra1*^Δbeta^ mice. Immunostaining for Ki67 staining revealed significantly fewer proliferating β-cells (Ki67^+^) in *Glra1*^Δbeta^ mice compared to the control mice. Conversely, staining for CC3 showed a substantial increase in apoptotic β-cells in the *Glra1*^Δbeta^ pancreas (Fig. 6a–d), demonstrating enhanced β-cell death. Notably, glycine administration attenuated Tg-suppressed β-cell proliferation (Fig. 6e). These findings indicate that GLRA1 signaling is essential for maintaining β-cell homeostasis by supporting proliferation and protecting against stress-induced apoptosis, thereby preserving β-cell mass during metabolic stress.

To assess whether glycine–GLRA1 signaling preserves β-cell function in a setting of β-cell loss, we used a streptozotocin (STZ)-induced model of insulinopenic diabetes. Repeated STZ administration selectively ablated pancreatic β-cells, resulting in markedly reduced circulating insulin levels under fed conditions, confirming severe β-cell dysfunction. In this type 1 diabetes (T1D) model, ­glycine supplementation failed to lower blood glucose or improve glucose tolerance, indicating that the effects of glycine require the presence of functional β-cells. Similarly, glycine treatment had no beneficial impact on glucose tolerance in β-cell-specific *Glra1* knockout mice (Fig. 6f–h;  Supplementary Fig. S9i). These findings demonstrate that glycine–GLRA1-mediated regulation of glucose homeostasis depends on preserved β-cell mass and function, further emphasizing that glycine acts via a β-cell-intrinsic signaling mechanism rather than through peripheral glucose metabolism.

### GLRA1 genetic variants and expression in human β-cells are associated with T2D

To evaluate the relevance of GLRA1 in human β-cell function and diabetes, we analyzed publicly available genetic and transcriptomic datasets. Using the Type 2 Diabetes Knowledge Portal, we identified “*very strong*” evidence (HuGE score = 45) for an association between GLRA1 and T2D at the gene level, integrating data from both common and rare variant analyses. Common variants in GLRA1 showed significant associations with acute insulin response (*P *= 9.96 × 10^−5^) and HbA1c levels (*P *= 2.12 × 10^−4^), while rare variants were also associated with HbA1c (*P *= 0.021) and 2-h postprandial glucose (*P *= 0.025; Supplementary Fig. S10a), implicating GLRA1 in insulin secretion and glycemic control. Transcriptomic analyses further supported these associations. Bulk RNA sequencing (RNA-seq) revealed significantly lower *Glra1* expression in pancreatic islets from individuals with T2D compared to non-diabetic controls (Supplementary Fig. S10c and d). Single-cell RNA-seq (scRNA-seq) confirmed that GLRA1 was downregulated specifically in β-cells from T2D donors (Supplementary Fig. S10e). Together, these genetic and expression data establish GLRA1 as a key regulator of human β-cell function and glucose homeostasis.

In summary, we identify a glycine–GLRA1–calmodulin signaling axis that regulates ER calcium homeostasis, alleviates ER stress, and promotes insulin biosynthesis and secretion in β-cells (Fig. 6i). These findings position GLRA1 as a critical β-cell regulator with both mechanistic and translational significance, and suggest that pharmacological activation of GLRA1 or dietary glycine supplementation may represent novel therapeutic strategies for the treatment of hyperglycemia in diabetes.

## Discussion

While glycine induces Cl^−^ currents in β-cells to promote membrane depolarization and Ca^2+^ influx [18], our findings reveal a complementary mechanism through GLRA1-driven regulation of ER calcium. Using β-cell-specific *Glra1* knockout models and ER-targeted calcium sensors, we show that GLRA1 activation sustained ER calcium levels, attenuated ER stress, and promoted insulin gene transcription. These observations are consistent with transcriptomic analyses in human islets, where GLRA1 expression is reduced in T2D, and genetic studies linking GLRA1 variants with glycemic traits [3].

Unlike its inhibitory role in the nervous system, glycine acts as a β-cell signal to regulate insulin production via GLRA1. Under metabolic stress, β-cells adapt by proliferating or expanding islet mass [37]. In mice with elevated glycine—through global *Shmt2* overexpression—we observed increased islet number and size, suggesting that glycine promotes β-cell proliferation and/or survival via GLRA1. Conversely, *Glra1* deletion phenocopied glycine deficiency, leading to increased ER stress, diminished islet mass, and heightened β-cell apoptosis. Since both serine and glycine were depleted in glycine-deficient diet, the contribution of serine to insulin production cannot be fully excluded. Additionally, glycine modulates glucagon secretion [38], suggesting that glycine may operate on other cell types within the islet to alter the paracrine regulatory network.

ER calcium homeostasis mitigates ER stress, a process critical for triggering insulin secretion [36, 39]. We show that GLRA1 interacts with calmodulin–IP_3_R, a known regulator of ER calcium dynamics [40]. Glycine strengthens this interaction, and calmodulin suppression abrogates glycine-induced ER calcium elevation and insulin secretion. We further demonstrate that glycine rescues Tg-induced ER calcium depletion and ER stress, likely through enhanced SERCA activity [41]. While our data support a SERCA-mediated mechanism, contributions from the ryanodine receptor (RyR)-mediated calcium release cannot be excluded. Although energy is not essential for glycine-mediated insulin secretion, ATP-dependent and mechano-sensing mechanisms may fine-tune calcium influx [42, 43], which could be attributed to GLRA1-enhanced calcium flux. Although calcium regulates insulin vesicle exocytosis, and calmodulin serves as a key regulator [44], the role of calmodulin in GLRA1-mediated enhancement of GSIS may extend beyond its canonical functions in calcium mobilization and ER stress attenuation.

In summary, our study uncovers a glycine–GLRA1–calmodulin signaling axis that regulates ER calcium homeostasis in pancreatic β-cells. Through this pathway, glycine enhances ER calcium levels, alleviates ER stress, and promotes insulin biosynthesis and secretion. By defining the molecular link between extracellular glycine sensing and intracellular calcium regulation, we establish a critical mechanism that sustains β-cell function and glucose homeostasis. These findings highlight GLRA1 as a potential therapeutic target and support glycine-based interventions for diabetes management. Moreover, our findings add to a growing body of evidence that amino acids function as signaling metabolites. Like leucine and glutamine, which act through the mammalian target of rapamycin (mTOR) and G protein-coupled receptors (GPCRs), respectively, glycine signals through the ionotropic receptor GLRA1 to directly regulate β-cell function. This highlights an emerging paradigm in which nutrient-receptor axes modulate endocrine cell biology.

### Limitations of the study

This study has several limitations. First, we focused on β-cell-mediated insulin secretion and did not explore potential effects of glycine on α-cell glucagon regulation. Second, glycine also acts as an N-methyl-D-aspartate (NMDA) receptor (NMDAR) co-agonist, which may independently modulate insulin secretion [45]; we ­cannot fully exclude the contributions of NMDAR. Third, although GLRA1 is expressed in macrophages [46] and macrophages influence β-cell development [47], the role of glycine in immune–islet crosstalk remains unaddressed. Finally, the molecular details of GLRA1–calmodulin interaction and ER calcium regulation *in vivo* warrant further mechanistic investigation. While our study demonstrates the glycine–GLRA1–calmodulin mechanism in mouse models and β-cell lines, a key future direction will be to directly recapitulate these findings in human islets. Confirming that glycine sustains ER calcium levels and reduces ER stress in a GLRA1-dependent manner in human β-cells will be a critical step in translating these discoveries into clinical relevance for human diabetes. While our data demonstrate that the glycine–GLRA1 axis is critical for β-cell function and whole-body glucose homeostasis, a future direction will be to investigate whether this pathway also influences peripheral glucose uptake in tissues such as the muscle and adipose.

## Materials and methods

### Human study and glycine measurement

#### The NSPT cohort

The NSPT cohort consists of 1027 Han Chinese volunteers recruited from the Chinese city Zhengzhou, Henan province by NSPT [24]. NSPT is a sub-project of the National Science & Technology Basic Research Project approved by the Ethics Committee of Human Genetic Resources of the School of Life Sciences, Fudan University, Shanghai (14117).

The plasma glycine levels of NSPT individuals were detected using Nuclear Magnetic Resonance (NMR) Spectroscopy. Each plasma sample (350 μL) was mixed with 350 μL phosphate buffer (0.085 mmol/L, pH 7.40, containing 10% D_2_O) and 600 μL mixture was transferred into a 5-mm NMR tube [48]. Samples were automatically handled at 7 °C using a SampleJetTM (Bruker Biospin, Germany) sample changer before NMR analysis. All NMR spectra were acquired at 310 K on a Bruker AVANCE III 600 MHz NMR spectrometer (600.13 MHz for 1H frequency) equipped with a BBI probe (Bruker Biospin, Germany) [49]. For each plasma sample, three spectra were collected, including one with the standard NOESYGPPR1D sequence (RD − 90°−t1 − 90°−tm − 90°−acq), a t2-edited spectrum with a standard Carr−Purcell−Meibom−Gill (CPMG) sequence (RD − 90°−(τ − 180°−τ)n−acq) with *τ* of 350 μs and *n* of 128, and a diffusion-edited spectrum (with the sequence, RD − 90°−G1−τ − 180°−G2−τ − 90°−Δ − 90°−G3−τ − 180°−G4−τ − 90°−te − 90°−acq) with Δ of 116 ms, *τ* of 200 μs, and te of 4.4 ms. While NOESYGPPR1D spectra contained all detectable signals of organic metabolites, CPMG spectra contained mainly signals from small metabolites or moieties with fast motions. In contrast, diffusion-edited lipid contained mainly signals from moieties in lipoproteins and acetyl-glycoproteins. The 90° pulse length was adjusted to about 10 μs for each sample and 32 transients were collected into 98 k data points over a spectral width of 30 ppm. For 1H NMR spectra, an exponential window function was employed with a line broadening factor of 0.3 Hz and zero-filled to 128 k prior to Fourier transformation. Glycine (δ3.56, singlet) and other 40 metabolites were quantified using the Bruker IVDr Lipoprotein Subclass Analysis B.I.LISA™ software package (Bruker Biospin, Germany) [50].

#### The ChiHOPE cohort

The ChiHOPE cohort consists of 835 drug-naïve patients with T2D recruited from two multi-center, randomized, double-anonymized phase 3 trials of chiglitazar (CMAP, placebo-controlled, ClinTrials.gov registration no. NCT02121717; [51] CMAS, sitagliptin-controlled, ClinTrials.gov registration no. NCT02173457) [52] (fudan-pgx.org/chihope/). Ethical approvals were obtained from Ethical Committees of the study centres (CMAP, *n *= 26; CMAS, *n *= 33). All procedures performed in the study involving human participants were in accordance with the ethical standards of the institutional and (or) national research committee, as well as with the Declaration of Helsinki and its later amendments or comparable ethical standards. All participants provided written informed consent.

The plasma glycine levels of ChiHOPE individuals were detected by Liquid Chromatography-Mass Spectrometry (LC-MS/MS). The metabolomics analysis was performed by Q300 Kit (Metabo-Profile, Shanghai, China). Plasma samples were prepared following the manufacturer’s instructions. Targeted metabolic profiling was conducted at Metabo-Profile (Shanghai, China) using an ultra-performance liquid chromatography coupled to tandem mass spectrometry (UPLC-MS/MS) system (ACQUITY UPLC-Xevo TQ-S, Waters Corp., Milford, MA, USA). The optimized instrument settings are as follows: for UPLC, column: ACQUITY UPLC BEH C18 1.7 × 10^−6^ m VanGuard precolumn (2.1 × 5 mm) and ACQUITY UPLC BEH C18 1.7 × 10^−6^ m analytical column (2.1 × 100 mm), column temp.: 40 °C, sample manager temp.: 10 °C, mobile phases: A = water with 0.1% formic acid; and B = acetonitrile/IPA (90:10), gradient conditions: 0–1 min (5% B), 1–12 min (5%–80% B), 12–15 min (80%–95% B), 15–16 min (95%–100% B), 16–18 min (100% B), 18–18.1 min (100%–5% B), 18.1–20 min (5% B); flow rate: 0.40 mL/min; and injection volume: 5.0 µL. For MS, capillary: 1.5 kV (ESI+), 2.0 kV (ESI−); source temp.: 150 °C; desolvation temp.: 550 °C; and desolvation gas flow: 1000 L/h. The raw data generated by UPLC-MS/MS were processed using the QuanMET software v2.0 (Metabo-Profile, Shanghai, China) to perform peak integration, calibration, and quantitation for each metabolite.

#### Mouse models

All mouse strains were obtained from GemPharmatech or The ­Jackson Laboratory. Experiments utilized male and female WT C57BL/6J, *Glra1*^fl/fl^ (GemPharmatech: Stock No. T059670), and *Ins2*-Cre (Jackson Laboratory: Stock No. 003573) mice. β-cell-specific *Glra1* knockout mice (*Glra1*^Δbeta^) were generated by crossing *Glra1*^fl/+^,*Ins2*-Cre males with *Glra1*^fl/+^ females, yielding littermates expressing or ­lacking *Glra1* in β-cells. *Glra1* knockout was confirmed via immunoblotting analysis of isolated islets. Mice were housed under specific pathogen-free conditions (20–22°C, 40%–60% humidity, 12-h light/12-h dark cycle) with *ad libitum* access to a standard chow diet (Research Diets, D10001). Customized HFDs—amino acid control (Cont. HFD, A20101501) and glycine/serine-deficient (ΔGS HFD, A22051101)—were formulated by Jiangsu Synergy Pharmaceutical Biological Co., Ltd, based on the D12492 composition (Supplementary Table S1). A commercial HFD (D12492) was purchased from Research Diets. For hyperglycemia models, 4–5-week-old mice were fed HFD or customized diets for 6–12 weeks. All strains were maintained on a C57BL/6J background, and mice were randomized to experimental groups by body weight. All procedures were approved by the Institutional Animal Care and Use Committee of Fudan University (Protocol ID: IDM2024015a) and conducted in compliance with institutional guidelines.

#### Materials and plasmids

Glycine (G8790), dextrose (D9434), and Polybrene (H9268) were ­purchased from Sigma. DAPI (C1002), Hoechst 33342 (C1028), and ATP determination kit (S0027) were obtained from Beyotime ­Biotechnology, Mag-Fluo-4-AM (20401) from AAT Bioquest, ­Bitopertin (HY-10809), W7 (HY-100912), Fluo-4AM (HY-101896) from MedChemExpress, streptozocin (STZ, A610130) from Sangon Biotech, insulin from Novo Nordisk, glucose test strips from Bayer, ultra-sensitive mouse insulin ELISA kits (MS100) from EZassay Biotechnology, glycine determination ELISA kits (CES117Ge) from Cloud-Clone Corp, thapsigargin (GC11482), carbamylcholine (GC16937) from GlpBio, and strychnine from local commercial vendors.

The following vectors were constructed using standard ­molecular cloning approaches: pAAV-CMV-*Shmt*2, pAAV-DIO-*Shmt*2, pLKO.1-shScramble, pLKO.1-sh*Glra1*, pLKO.1-sh*Glyt1*, and pLKO.1-sh*Glyt2*. Packaging plasmids psPAX2 (Addgene #12260) and pMD2.G (Addgene #12259) were obtained from Addgene, while pHelper (P0243), pAAV-RC9 (P2846), and pAAV-CMV-NPY-EGFP (P58784) were purchased from Miaoling Biotechnology. ER calcium sensor CEPIA1er was obtained from Miaoling plasmid platform.

#### Cultured cell models and gene silencing

HEK293T cells were cultured in high-glucose Dulbecco’s modified Eagle’s medium (DMEM; 12800082, Gibco) supplemented with 10% (v/v) fetal bovine serum (FBS; F7524, Sigma), 100 U/mL penicillin, and 100 μg/mL streptomycin (C0222, Beyotime) at 37 °C under 5% CO_2_. INS1 cells (kindly provided by Dr. Yan Chen, Chinese Academy of Sciences) were maintained in RPMI-1640 medium containing 10 mmol/L HEPES, 100 U/mL penicillin, 100 μg/mL streptomycin (C0222, Beyotime), 10% FBS, and 50 μmol/L β-mercaptoethanol (21985023, Gibco). *Glra1* and *Glyt1* were knocked down in INS1 cells using a lentiviral shuttle plasmid (pLKO.1-TRC) expressing shRNA targeting the respective genes. Polybrene (10 μg/mL; 107689, Sigma) was added to enhance lentiviral transduction efficiency during cell seeding, and experiments were conducted 48 h post-infection. Knockdown efficiency was validated by western blotting or quantitative PCR (qPCR).

#### Lentivirus preparation for gene silencing

HEK293T cells were grown in gelatin-coated 100-mm dishes and cotransfected with three plasmids: 5 μg shuttle plasmid, 3 μg psPAX2 packaging plasmid, and 2 μg pMD2.G envelope plasmid using PEI MAX transfection reagent (1 mg/mL, pH 7.0; Polysciences, 24765). At 6 h posttransfection, the medium was replaced with high-glucose DMEM supplemented with 30% (v/v) FBS to enhance lentivirus production. Viral particles were harvested at 48 h and 72 h posttransfection, pooled, and filtered through a 0.45-μm pore-size membrane (Jet Biofil) to remove cellular debris. Lentiviral particles were concentrated via ultracentrifugation (25,000 rpm, SW 41 rotor, 90 min, 4 °C; Beckman Coulter). The pellet was resuspended in Dulbecco’s phosphate-buffered saline (DPBS; Boster Biological Technology) overnight at 4 °C, aliquoted into 20 μL, and stored at −80°C. shRNA sequences targeting rat *Glra1* (5′-GTGTCCTACGTGAAAGCTATT-3′), *Glyt1* (5′-ATGGCATCCTACAACAAATTC-3′), and *Calm* (5′-ACGCTGTGTTCTTTGCATTTG-3′) were cloned into the pLKO.1-TRC vector for lentiviral knockdown constructs.

#### AAV2/9-mediated gene expression

AAVs were packaged using HEK293T cells cultured on gelatin-coated 150-mm plates at 70%–80% confluency. For transfection, 30 μg pHelper plasmid, 15 μg AAV9 packaging plasmid, and 15 μg AAV2 transfer plasmid (carrying the gene of interest) were mixed with 180 μL PEI MAX transfection reagent (24765, Polysciences) in 1.5 mL Opti-MEM (Gibco). After vortexing and 15-min incubation at room temperature (RT), the mixture was added dropwise to cells and incubated for 6 h. Cells were harvested at 72 h post-transfection, lyzed in buffer containing 0.5% sodium deoxycholate and 50 U/mL benzonase (C2001, HaiGene Biotech), and purified via iodixanol density-gradient ultracentrifugation (OptiPrep, D1556, Sigma) at 50,000 rpm (Type 70Ti rotor, 1 h, 18 °C). The viral fraction (40%–60% iodixanol) was collected, concentrated using an Amicon Ultra-15 filter (UFC901008, 100 kDa cutoff, Millipore), and titrated by qPCR using ITR- or WPRE-specific primers (ITR-F: 5′-GCGTCGGGCGACCTTTGGT-3′, ITR-R: 5′-AGGAACCCCTAGTGATGGAG-3′; WPRE-F: 5′-CCCTCCCTATTGCCACGGCG-3′, WPRE-R: 5′-AGAAGGACGTCCCGCGCAGA-3′).

AAV2/9 vectors encoding SHMT2 or the site-selective recombination FLEx switch vector (AAV-FLEx), which carried the reverse open reading frame harboring mouse *Shmt*2, were intravenously (IV) administered to 4–6-week-old mice at a dose of 5 × 10^11^ genomic copies (GC) in 100 μL normal saline. Protein levels in the liver or pancreatic islet tissues were validated by immunoblotting analysis 2 weeks after AAV administration. AAV-GFP served as the control. In studies using the AAV-FLEx vector, WT mice injected with AAV-FLEx were employed as controls for comparison with *Ins2*-Cre-expressing mice.

#### GTT, ITT, and PTT

For GTT, mice were fasted for 6 h (9:00 a.m. to 3:00 p.m.) in cages with sawdust bedding and injected intraperitoneally (i.p.) with glucose (1 g/kg body weight) [53]. Blood glucose levels were measured at 0, 15, 30, 60, 90, and 120 min post-injection using a Contour Plus glucometer (Bayer). For ITT, mice were fasted for 4 h (10:00 a.m. to 2:00 p.m.) and received intraperitoneal insulin (1 U/kg). Blood glucose was assessed at 0, 15, 45, 90, and 120 min. For PTT, mice were fasted overnight to deplete hepatic glycogen, followed by intraperi­toneal injection of pyruvate (1 g/kg), with glucose measured at 0, 15, 30, 60, 90, and 120 min.

In STZ-induced diabetes experiments, tail-vein blood was collected, and glucose levels were quantified colorimetrically using PGO enzymes (P7119, Sigma) coupled with o-dianisidine (F5803, Sigma) on a Spark multimode microplate reader (Tecan). For all metabolic tests (GTT, ITT, and PTT), the area under the curve (AUC) was calculated relative to the baseline (*t* = 0).

#### STZ-induced T1D mouse model

Mice were fasted for 4 h (9:00 a.m. to 1:00 p.m.) and then i.p. injected with freshly prepared STZ at a dose of 50 mg/kg body weight. STZ was dissolved at 5 mg/mL in 0.1 mol/L sodium citrate buffer (pH 4.5), prepared by dissolving 1.47 g sodium citrate in 50 mL distilled water and adjusting the pH with 0.1 mol/L citric acid. The solution was sterilized by filtration (0.22 μm) and administered within 15–20 min of preparation to ensure stability. Mice received daily injections for 5 consecutive days. After the final STZ injection, mice were randomly assigned to two groups: one group received standard drinking water, while the other was provided *ad libitum* access to 1% (w/v) glycine dissolved in sterile-filtered water for an additional 2 weeks. Blood glucose and body weight were monitored weekly to assess diabetic phenotype progression.

#### Body composition and metabolic cage system

Body composition was analyzed using a Bruker Minispec LF50 live mouse analyzer. Mice were individually weighed and gently restrained in a thin-walled plastic holder for measurement of lean and fat mass, following the manufacturer’s protocol. EE was assessed using a metabolic cage system (CLAMS-16M, Columbus Instruments) under controlled conditions (12-h light/12-h dark cycle [7:00 a.m. to 7:00 p.m.], 20–22°C, *ad libitum* food/water). After a 12-h acclimation period, data were collected continuously for 72 h using Oxymax software for instrument control and acquisition. EE was calculated via the Weir equation, and the respiratory quotient was derived as the ratio of carbon dioxide production to oxygen consumption (VCO_2_/VO_2_).

#### Islet isolation and GSIS assays

Mice were euthanized, and the peritoneal cavity was surgically exposed to clamp the pancreatic duct at its intestinal junction. The common bile duct was cannulated *in situ*, and the pancreas was distended by retrograde injection of 2 mL freshly prepared collagenase XI solution (0.65 mg/mL in HBSS supplemented with 0.1% BSA; Collagenase IV, Labelead) using a 3-mL syringe. The dissected pancreas was incubated in a 37 °C water bath for 15 min to facilitate enzymatic digestion. Following digestion, the tissue was mechanically dispersed by vigorous shaking and filtered through a sterile mesh to remove cellular debris. The resulting suspension was centrifuged briefly (300 *g*, 2 min), and the pellet was resuspended in 10 mL Histopaque 1119 (1119-1, Sigma). A discontinuous density gradient was prepared by layering 6 mL Histopaque 1077 (1077-1, Sigma) beneath the Histopaque 1119-islet mixture, followed by 6 mL HBSS containing 0.1% BSA. The gradient was centrifuged at 339 *g* for 25–30 min at 4 °C. Pancreatic islets were harvested from the interface between the Histopaque 1077 and HBSS layers, yielding approximately 200 islets per mouse. Islets were washed three times in HBSS containing 0.1% BSA, manually purified by hand-picking, and subsequently cultured in RPMI 1640 medium supplemented with 10% FBS at 37 °C in a humidified 5% CO_2_ incubator.

Twenty pancreatic islets in each 1.5-mL tube were washed twice with glucose-free Krebs-Ringer HEPES buffer (20 mmol/L HEPES, 136 mmol/L NaCl, 4.7 mmol/L KCl, 1 mmol/L MgSO_4_, 1 mmol/L CaCl_2_, and 5 mmol/L KH_2_PO_4_, pH 7.4). Islets were subsequently starved for 1 h at 37 °C in 500 μL Krebs-HEPES buffer containing 2.8 mmol/L glucose, with or without additional stimuli. Insulin secretion was assessed sequentially under three conditions: low glucose (LG: 2.8 mmol/L glucose), high glucose (HG: 16.7 mmol/L glucose), and depolarization with potassium chloride (KCl: 30 mmol/L), each for 20 min at 37 °C. The supernatant (200 μL per condition) was collected and stored at −80°C until insulin quantification using a commercial ELISA kit (CES117Ge, Cloud-Clone Corp.), following the manufacturer’s protocol. After sti­mulation, islets were lyzed in acid-ethanol (75% ethanol and 0.18 mol/L HCl) containing Proteinase K for genomic DNA extraction (DC102, Vazyme Biotech). DNA concentration was quantified using a NanoDrop spectrophotometer (Thermo Fisher Scientific).

INS1 cells were validated for insulin expression and glucose-responsive secretion. Mycoplasma contamination testing confirmed cell line integrity. Cells were seeded onto gelatin-coated 24-well plates and grown to 80% confluency prior to experiments. GSIS assays were performed as previously described [54]. For glycine/serine deprivation studies, INS1 cells or isolated islets were preconditioned for 24 h in glycine/serine-depleted RPMI 1640 medium (Gibco), followed by insulin secretion assessment in Krebs-HEPES buffer. Secreted insulin in supernatants was quantified via ELISA, and results were normalized to total cellular DNA content.

#### Blood insulin and lipid measurement

Mice were fasted for 4 h (9:00 a.m. to 1:00 p.m.) and administered an intraperitoneal injection of glucose (1 g/kg body weight). Blood samples were collected via tail-vein bleeding at baseline (0 min, pre-injection) and at 10 and 30 min post-injection. Plasma insulin levels were quantified using an Ultra-Sensitive Mouse Insulin ELISA kit (CES117Ge, Cloud-Clone Corp.) according to the manufacturer’s instructions.

For total cholesterol (TC) and TG measurements, mice were fasted for 4–6 h, and blood was collected via retro-orbital plexus sampling under isoflurane anesthesia. Plasma was isolated by centrifugation (2000 *g*, 10 min, 4 °C) and stored at −80°C until analysis. TC and TG concentrations were determined using commercial enzymatic assay kits (TC: #A111-2-1; TG: #A110-2-1; Nanjing Jiancheng Bioengineering Institute) following standardized protocols.

#### Calcium signaling determination

INS2 cells were seeded in 4-well chambered glass-bottom dishes (Cellvis) and transduced with lentivirus encoding the ER-targeted calcium sensor CEPIA1er [31, 32] for 48 h. Cells were incubated in the medium supplemented with a 2% lipid mixture (L0288, Sigma) to resemble HFD condition *in vivo* before the assays. To monitor ER calcium dynamics in islets from HFD-fed mice, isolated islets were incubated with 20 μmol/L Mag-Fluo-4 AM for 60 min at 37 °C, followed by permeabilization and a 30-min dye equilibration period. For the cytosolic calcium determination, cells were treated with 2 μmol/L Fluo-4AM for 30 min at 37 °C, followed by washing with HEPES buffer for three times. Fluorescence intensity was subsequently recorded at 0.7-s intervals using a Tecan plate reader (Ex/Em 488 nm/520 nm) [32, 55]. For knockdown experiments, cells were co-transduced with shRNA targeting specific genes. Before imaging, cells were washed with PBS and equilibrated for 1 h at 37 °C in HEPES buffer (5 mmol/L HEPES, 121 mmol/L NaCl, 4.7 mmol/L KCl, 1.2 mmol/L MgSO_4_, 1.2 mmol/L KH_2_PO_4_, 5 mmol/L NaHCO_3_, 2.0 mmol/L CaCl_2_, and 1 mmol/L glucose, pH 7.4). During equilibration, pharmacological agents were added: glycine (1 mmol/L), strychnine (5 μmol/L), or Tg (1 μmol/L). For glycine/serine deprivation studies, cells were preconditioned for 24 h in the glycine/serine-deficient RPMI 1640 medium (Gibco) before equilibration in the HEPES buffer. Live-cell imaging was performed on a Nikon A1R HD confocal microscope equipped with a 60× oil-immersion objective (Ex/Em 488 nm/520 nm, exposure time 982 ms). Baseline fluorescence (F_0_) was recorded for 5 min, followed by the addition of stimuli (in HEPES buffer) and continuous image acquisition for 25 min. Fluorescence intensity was quantified using ImageJ (NIH), and data were expressed as the normalized ratio F/F_0_.

#### Immunofluorescence microscopy

Dissected tissues were fixed in 4% paraformaldehyde for at least 24 h, washed with PBS, and dehydrated in 30% sucrose overnight. Tissues were embedded in Optimal Cutting Temperature (OCT) compound, processed for cryosectioning at 8-μm thickness, and mounted onto adhesive slides (188105, Citotest). For immunostaining, sections were blocked with 5% goat serum in PBS and incubated overnight at 4 °C with primary antibodies: anti-insulin (1:500, 66198-1-Ig, Proteintech), anti-glucagon (1:200, A22702, ABclonal), and anti-Ki67 (1:200, A20018, ABclonal). For BIP staining, INS2 cells grown on collagen-coated coverslips were treated with Tg (1 μmol/L) and/or glycine (50 mmol/L), fixed, and stained with anti-BIP antibody (1:100, 11587-1-AP, Proteintech). Secondary antibodies, Alexa Fluor 488-conjugated anti-mouse (1:200, A-11001, Invitrogen) and Alexa-Fluor-647-anti-rabbit secondary antibody (1:200, A-21244, Invitrogen), were applied for 1–2 h at RT. Nuclei were counterstained with DAPI (C1002, Beyotime). Stained sections were imaged using an Evident Olympus VS200 slide scanner with a 20× objective. Quantitative analysis was performed in OlyVIA software: islets were manually annotated, insulin/glucagon-positive cells were classified based on fluorescence intensity thresholds (≥ 3× background), and the ratio of glucagon^+^ cells to total DAPI^+^ nuclei within annotated islets was calculated. Methods for immunostaining were adapted from previous work [54].

Insulin granule docking was visualized in INS1 cells expressing the granule marker NPY-EGFP using total internal reflection fluorescence (TIRF) microscopy [56]. Imaging was performed on a Nikon Ti-E inverted microscope equipped with a 100× objective. A 488-nm laser was used for excitation with the TIRF angle set to 61.6°, yielding an estimated evanescent field penetration depth of approximately 100 nm. For each experimental condition, time-lapse sequences of at least 40–50 randomly selected cells were acquired at 100-ms intervals for a minimum duration of 40 s. Exocytosis events were manually identified by the characteristic rapid disappearance of granule fluorescence within 1–2 frames. Granules that moved into the evanescent field were classified as docking events, and the number of docked granules was quantified using the “find maxima” function in ImageJ.

#### H&E staining and IHC

Tissues were routinely processed for paraffin embedding, sectioned at 3-μm thickness, and mounted on glass slides. For select expe­riments, cryosectioning was performed as previously described. Hematoxylin and eosin (H&E) staining of adipose and liver sections was conducted using a commercial kit (BL700B, Biosharp) following the manufacturer’s protocol. Liver cryosections were stained with Oil Red O (O0625, Sigma) to visualize lipid droplets, while Masson’s trichrome staining (BSBA-4079A, ZSGB-BIO) was applied to paraffin-embedded sections to assess collagen deposition.

For insulin immunohistochemistry (IHC), pancreatic sections were incubated with anti-insulin primary antibody (1:500, 66198-1-Ig, Proteintech), followed by incubation with HRP-conjugated anti-mouse IgG antibody (1:200, RT, 1–2 h), and developed with DAB substrate (ZLI-9018, ZSGB-BIO). Bright-field images were acquired at 20× magnification using an Olympus VS200 slide scanner. β-cell area was quantified via positive-pixel count analysis in OlyVIA software (Olympus). Islet number and size were determined by manually annotating insulin-positive clusters across entire pancreatic sections.

Adipocyte size was analyzed in H&E-stained white adipose tissue sections using ImageJ with the Adiposoft plugin (automated mode) to calculate individual adipocyte cross-sectional areas.

#### Quantitative RT-PCR

Total RNA was extracted using TRIzol Reagent (R401-01-AA, Vazyme Biotech) following the manufacturer’s instructions. cDNA was synthesized from 1 μg RNA using HiScript III RT SuperMix (R323, Vazyme Biotech). qPCR was performed in triplicate with ChamQ Universal SYBR qPCR Master Mix (Q711, Vazyme Biotech) on a QuantStudio 7 Real-Time PCR System (Applied Biosystems). Primer sequences for target genes were designed and are listed in Supplementary Table S2.

### Co-immunoprecipitation and immunoblot analyses

INS2 cells transduced with lentiviral particles encoding HA-tagged GLRA1 for 48 h were stimulated overnight with a medium containing glycine (0, 0.133 mmol/L, or 5 mmol/L). Cells were lyzed on ice for 1 h in RIPA buffer (50 mmol/L Tris-HCl, 150 mmol/L NaCl, 1% NP-40, 0.5% sodium deoxycholate, and 0.1% SDS, pH 7.4) supplemented with protease inhibitor cocktail (MCE, #HY-K0010), with intermittent vortexing every 20 min. Lysates were centrifuged at 13,000 *g* for 10 min at 4 °C, and supernatants were incubated overnight at 4 °C with anti-GLRA1 antibody (1:100, 17951-1-AP, Proteintech), followed by incubation with protein A/G magnetic beads (SM005000, Smart-Lifesciences) for 2 h at RT. Beads were then washed three times with RIPA buffer, and bound proteins were eluted in 2× Laemmli SDS sample buffer (62.5 mmol/L Tris-HCl, 2% SDS, 10% glycerol, and 0.01% bromophenol blue, pH 6.8) at 95 °C for 5 min.

Protein lysates were resolved on 10% SDS-polyacrylamide gels (SDS-PAGE) at 100  V using a XinPRO-4 Mini Vertical Blot System (CLINX) and transferred to PVDF membranes (0.45 μm, Millipore) at 100  V for 1 h in transfer buffer (25 mmol/L Tris, 192 mmol/L glycine, and 20% methanol). Membranes were blocked for 1 h at RT in 5% (w/v) non-fat milk (Sangon Biotech) in PBS-T (PBS + 0.02% Tween-20, BBI). For phosphorylated proteins, blocking was performed with 3% (w/v) BSA (Sangon Biotech) in PBS-T. After blocking, membranes were incubated overnight at 4 °C with primary antibodies. Membranes were then washed three times in PBS-T and incubated with HRP-conjugated secondary antibodies (1:10,000, Jackson ImmunoResearch) in PBS-T for 1 h at RT. Signal detection was performed using the ChemiScope 6000 imaging system (CLINX) with enhanced chemiluminescence substrate. Band intensities were quantified using ImageJ (NIH).

The following antibodies were used in the study: anti-CHOP (15204-1-AP, Proteintech), anti-ATF4 (10835-1-AP, Proteintech), anti-BIP (11587-1-AP, Proteintech), anti-ATF6-p90 (24169-1-AP, ­Proteintech), anti-IRE1 (DF7709, Affinit Biosciences), anti-p-eIF2a (AF3087, Affinit Biosciences), anti-eIF2a (AF6087, Affinit ­Biosciences), anti-p-JNK (AF3318, Affinit Biosciences), anti-JNK(AF6318, Affinit Biosciences), anti-CC3 (25128-1-AP, Proteintech), anti-insulin (66198-1-lg, Proteintech), anti-GLRA1 (A2958, ABclonal), anti-SHMT2(33443S, CST), anti-calmodulin (28270-1-AP, Proteintech), and anti-calnexin (10427-2-AP, Proteintech).

### Bioinformatics analysis

To investigate the association between plasma glycine levels and diabetes traits, plasma glycine levels quantified by metabolomics from the NSPT and ChiHOPE cohorts were obtained. Generalized linear regression analyses were conducted with the *glm* function from the R package stats (v4.4.1). Comparisons among multiple groups were conducted with the *aov* function from the R package stats (v4.4.1), and *post-hoc* test were conducted with the *emmeans* function from the R package *emmeans* (v1.10.5). Partial correlation analysis was conducted using the *pcor* function from the R package ppcor (v1.1).

To compare GLRA1 expression in human islets between health and T2D patients, bulk gene expression data were accessed at Gene Expression Omnibus as GSE38642 [57] and GSE164416 [58]. Comparisons among groups were conducted with the *aov* function from the R package stats (v4.4.1), and *post-hoc* test were conducted with the *emmeans* function from the R package *emmeans* (v1.10.5). To determine GLRA1 expression in specific cell types, single-cell gene expression data were accessed at EMBL’s European Bioinformatics Institute (EMBL-EBI) as E-MTAB-5061 [15]. Genes with the highest biological variation from endocrine cells were obtained for the *t*-distributed stochastic neighbor embedding (*t*-SNE) analyses with *Rtsne* function from the R package Rtsne (v0.17). Analyses were conducted using R version 4.4.1 (2024-06-14).

### Human genetic association data

To identify gene- and variant-level associations with glycemic and related traits, we utilized the Type 2 Diabetes Knowledge Portal, a biomedical knowledge base for sharing genetic and genomic data. Gene-level associations were evaluated using Human Genetic Evidence (HuGE) Scores, which aggregate evidence from common variants (genome-wide association studies) and rare variants (exome-wide association studies) available in the portal. HuGE scores range from 1 (no evidence) to more than 350 (compelling evidence), with higher scores reflecting stronger genetic associations. For the evidence of rare variant, only analyses and burden tests were used.

### Statistical analysis

Sample sizes for animal studies were determined based on prior published methodologies and statistical power requirements. Data are presented as mean ± SEM. Comparisons between two experimental groups were analyzed using an unpaired two-tailed Student’s *t*-test unless specified. For comparisons involving three or more groups, one-way or two-way ANOVA followed by appropriate *post-hoc* multiple comparisons tests (e.g. Tukey’s or Sidak’s) was applied. Statistical significance was defined as *P *< 0.05. All experiments were independently replicated at least twice with consistent results. Ana­lyses were performed using GraphPad Prism 9 (GraphPad Software).

## Supplementary Material

loaf044_Supplementary_Data

## Data Availability

All the data supporting the findings of this study are available within the supplementary material and corresponding authors.
