# Supplementary material for: Glycine–GLRA1–calmodulin signaling regulates endoplasmic reticulum calcium to sustain insulin secretion and β-cell function
Source: Life Metab. 2025 Dec 19;5(2):loaf044. doi: 10.1093/lifemeta/loaf044 (PMC13110115; doi:10.1093/lifemeta/loaf044)
Supplement: loaf044_Supplementary_Data [file loaf044_supplementary_data.docx]

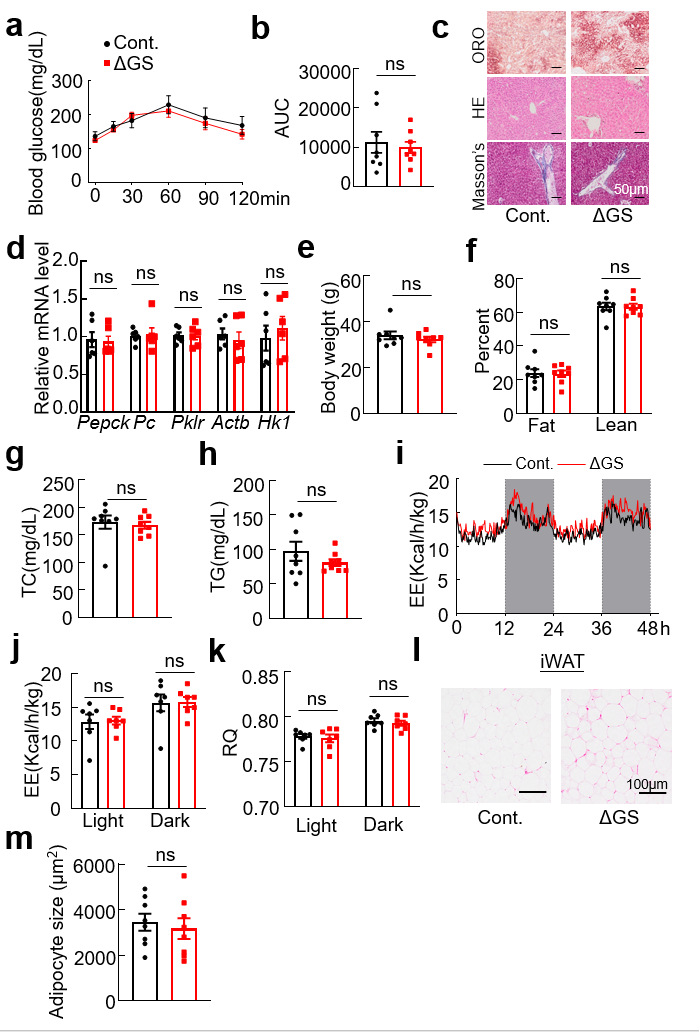


**Supplementary Figure S1** Glycine deficiency does not affect peripheral tissue metabolism, related to Figure 2. (a and b) PTT (a) and its quantification (b) in mice treated with glycine/serine-deficient (ΔGS) diet for 12 weeks. *n* = 8 for both groups. (c) Representative immunohistochemistry staining images of liver sections. *n* = 8 for both groups. Scale bar: 50 μm. (d) Hepatic gluconeogenesis mRNA levels. *n* = 6 for both groups. (e and f) Body mass (e) and percentage of fat mass and lean mass (f). *n* = 8 for both groups. (g and h) Serum total cholesterol (g) and triglycerides (h). *n* = 8 for both groups.. (i and j) Energy expenditure (i) and average 48-h energy expenditure quantification (j). *n* = 7 for both groups. (k) Quantification of respiratory quotient (RQ). *n* = 8 for both groups. (l and m) Representative H&E staining of white adipose tissue (l) and the quantification (m) of adipocyte size. *n* = 8 for both groups. Scale bar: 100 μm. Data are expressed as mean ± SEM. ns, not significant.


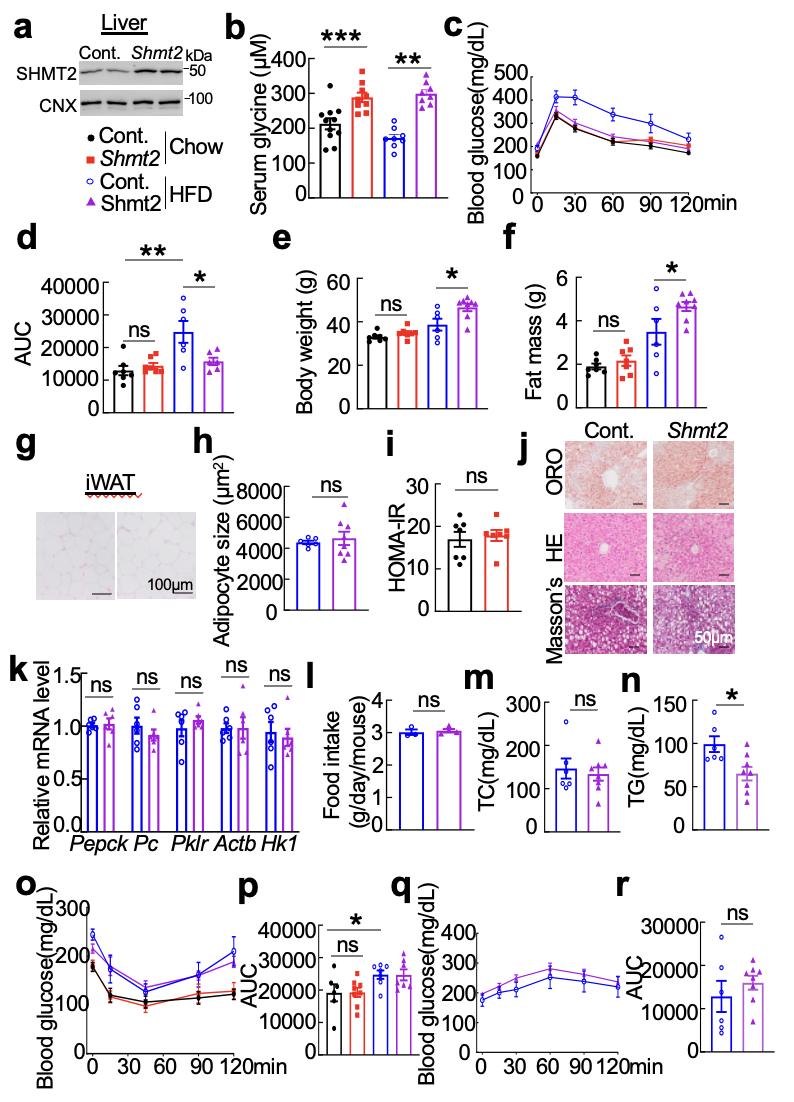


**Supplementary Figure S2** Genetically overexpression of *Shmt2* enhances glycine to improve glucose homeostasis, related to Figure 2. (a) Representative immunoblotting images of SHMT2 in the liver. The experiment was independently repeated for two times. (b) Serum glycine levels in mice overexpressing *Shmt2*. *n* = 11, 9, 8, and 8, respectively. (c and d) GTT (c) and its AUC quantification (d). *n* = 7, 7, 6, and 6, respectively. (e and f) Body mass (e) and fat mass (f). *n* = 7, 7, 6, and 8, respectively. (g and h) Representative images of white adipose tissue H&E staining (g) and the quantification (h) of adipocyte size. *n* = 6 and 8, respectively. (i) HOMA-IR in chow diet-fed mice overexpressing *Shmt2*. *n* = 7 in both groups. (j) Oil Red O, H&E, and Masson’s staining of the liver sections. *n* = 6 and 8, respectively. Scale bar: 50 μm. (k) Hepatic gluconeogenesis gene expression in mice overexpressing *Shmt2* globally. *n* = 6 in both groups. (l) Food intake. Each point represents two mice in each cage. *n* = 3 in both groups. (m and n) Serum total cholesterol (m) and triglycerides (n). *n* = 6 and 8, respectively. (o and p) ITT (o) and its AUC quantification (p). *n* = 7, 7, 6, and 8, respectively. (q and r) PTT (q) and its AUC quantification (r). *n* = 6 and 8, respectively. Data are expressed as mean ± SEM. ^*^*P* < 0.05; ^**^*P* < 0.01; ^***^*P* < 0.001; ns, not significant.


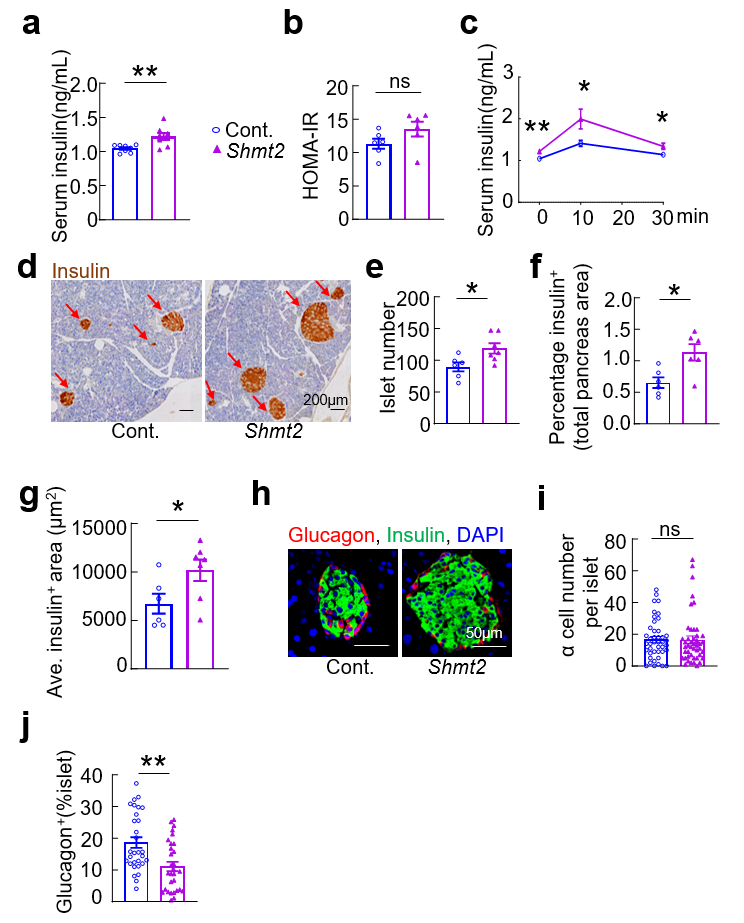


**Supplementary Figure S3** Ectopic SHMT2 expression increases insulin production, related to Figure 2. (a) Fasting blood insulin levels in HFD-fed mice overexpressing *Shmt2*. *n* = 8 in both groups. (b) HOMA-IR in HFD-fed mice overexpressing *Shmt2*. *n* = 6 in both groups. (c) GSIS in mice ectopically expressing *Shmt2*. *n* = 8 in both groups. (d−g) Pancreas insulin IHC staining (d), quantification of islet number per section (e), percentage of insulin-positive area relative to total pancreatic area (f), and the average insulin-positive area per islet (g). *n* = 6 in both groups. Red arrows indicate islets. Scale bar: 200 μm. Islet numbers and areas were counted from at least 10 sections. (h−j) Immunofluorescence images of insulin and glucagon staining in pancreas section (h), α-cell number per islet (i), and the quantification of glucagon-positive area (j). *n* = 30 islets from five mice per group. Scale bar: 50 μm. Data are expressed as mean ± SEM. ^*^*P* < 0.05; ^**^*P* < 0.01; ^***^*P* < 0.001; ns, not significant.


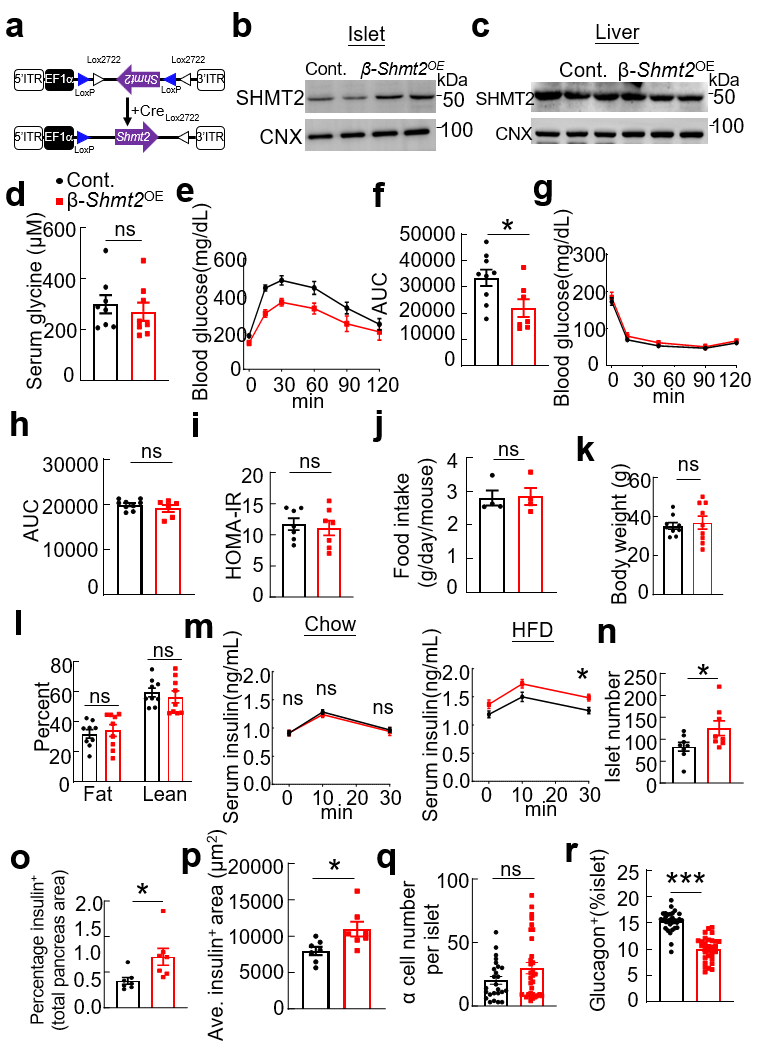


**Supplementary Figure S4** β-cell selective overexpression of *Shmt*2 on glucose homeostasis, related to Figure 2. (a) Schematic diagram of DIO-*Shmt*2 construction. (b and c) Representative immunoblotting images of SHMT2 in the islet (b) or the liver (c). The experiment was independently repeated for 2−3 times. (d) Serum glycine in mice overexpressing *Shmt*2 selectively in islets. *n* = 8 in both groups. (e and f) GTT (e) and its AUC quantification (f) in mice overexpressing *Shmt*2 in islets. *n* = 9 and 7, respectively. (g and h) ITT (g) and its AUC quantification (h) in mice overexpressing *Shmt*2 in the islets. *n* = 9 and 7, respectively. (i) HOMA-IR in mice overexpressing *Shmt*2 in the islets. *n* = 7 in both groups. (j) Food intake. Each point represents two mice in each cage. *n* = 4 in both groups. (k and l) Body mass (k) and percentage of fat and lean mass (l). *n* = 9 in both groups. (m) GSIS in chow diet (left) versus HFD-fed (right) mice overexpressing *Shmt*2 selectively in the islets. *n* = 7 in both groups. (n−r) Quantification of islet number per section (n), percentage of insulin-positive area relative to total pancreatic area (o), the average insulin-positive area per islet (p), α-cell number per islet (q), and the quantification of glucagon-positive area (r). *n* = 7 in both groups. Islet numbers and areas were counted from at least 10 sections. *n* = 30 islets from five mice per group that employed for the quantification of glucagon-positive area. *Ins2*-Cre male mice were i.v. injected with 5 × 10^11^ gc/mouse AAV-*Gfp* or AAV-DIO-*Shmt*2 and placed on D12492 diet for 12 weeks. Followed by GTT, ITT, PTT, and GSIS determination. Data are expressed as mean ± SEM. ^*^*P* < 0.05; ^**^*P* < 0.01; ^***^*P* < 0.001; ns, not significant.


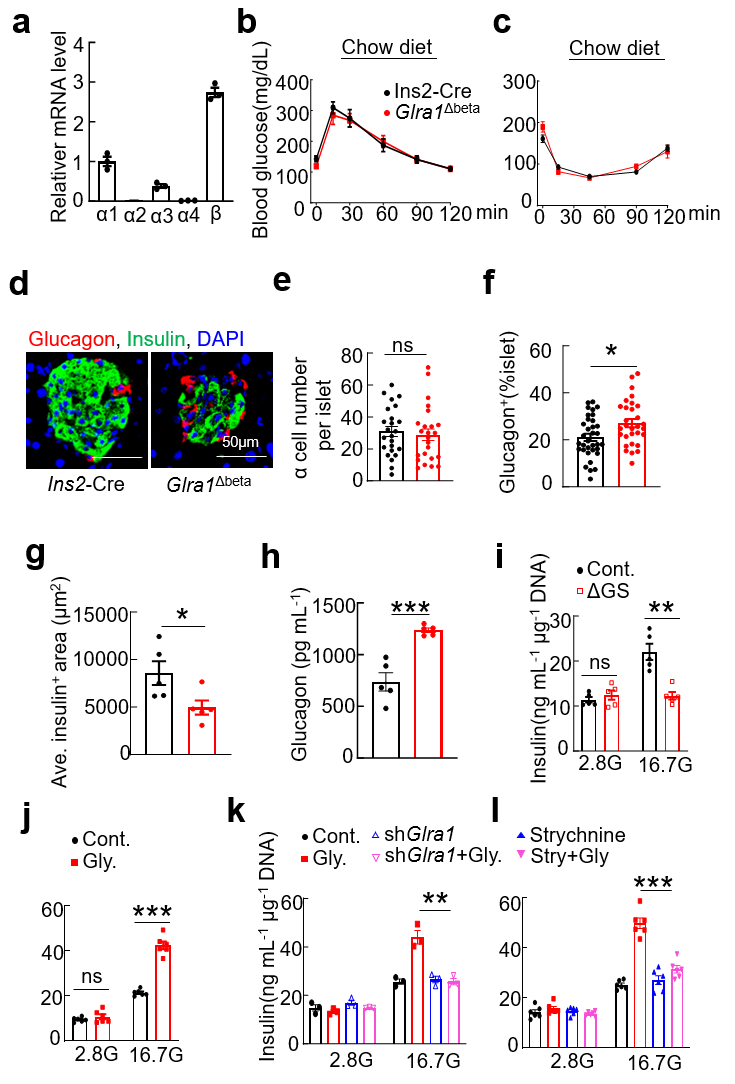


**Supplementary Figure S5** Glycine promotes insulin biosynthesis via GLRA1, related to Figure 3. (a) Expression of glycine receptor isoforms in the INS1 cells. *n* = 3. (b and c) GTT (b) and ITT (c) in chow diet-fed mice expressing or lacking *Glra1* in β-cells. *n* = 6 in both groups. (d−g) Immunofluorescence images of insulin and glucagon staining in pancreas sections (d), α-cell number per islet (e), quantification of glucagon-positive area (f), and the average insulin-positive area per islet (g) in HFD-fed mice. *n* = 30 islets from five mice per group. Scale bar: 50 μm. (h) Serum glucagon in HFD-fed mice expressing or lacking *Glra1* in β-cells. *n* = 5 in both groups. (i and j) GSIS in INS1 cells under conditions of glycine deficiency (i) or supplementation (10 mmol/L) (j). *n* = 5 for both groups in (i) or *n* = 6 for both groups in (j). (k and l) GSIS in INS1 cells under *Glra1* knockdown (k) or suppression with strychnine (3 μmol/L) (l) followed by glycine administration. *n* = 3 for all groups in (k); *n* = 6 for all groups in (l). Data are expressed as mean ± SEM. ^*^*P* < 0.05; ^**^*P* < 0.01; ^***^ *P* < 0.001; ns, not significant.


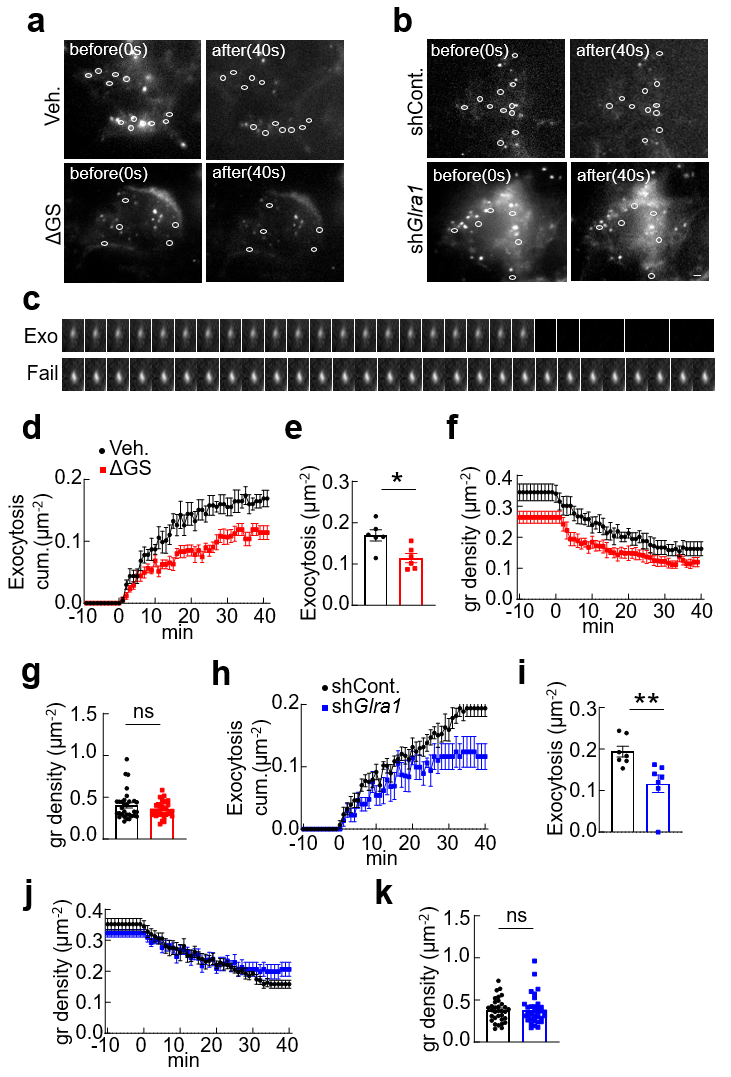


**Supplementary Figure S6** Glycine increases insulin granule docking to the plasma membrane via GLRA1, related to Figure 3. (a and b) Representative TIRF images of INS1 cells under the conditions of glycine deficiency (a) or *Glra1* knockdown (b), expressing NPY-EGFP before and after stimulation with 75 mmol/L K^+^ for 40 s. Scale bar: 1 μm. (c) Image sequence (0.1 s per frame) showing two individual granules in K^+^-stimulated control cells that underwent exocytosis (exo, top) or failed to do so (fail, bottom). (d−f) Cumulative exocytosis (d), quantification of the cumulative exocytosis at the end of 40-s K^+^ stimulation (e), and granule density (f) in cells cultured in glycine-deficient medium, stimulated with 75 mmol/L K^+^ from t = 0. *n* = 6 for both groups. (g) Average granule density in cells treated with vehicle or glycine-deficient medium. *n* = 31 and 33, respectively. (h−j) Cumulative exocytosis (h), quantification of the cumulative exocytosis at the end of 40-s K^+^ stimulation (i), and granule density (j) under GLRA1 deficiency, stimulated with 75 mmol/L K^+^ from t = 0. *n* = 6 for both groups. (k) Average granule density in cells expressing or lacking *Glra1*. *n* = 33 and 34, respectively. Data are expressed as mean ± SEM. ^*^*P* < 0.05; ^**^*P* < 0.01; ^***^*P* < 0.001; ns, not significant.


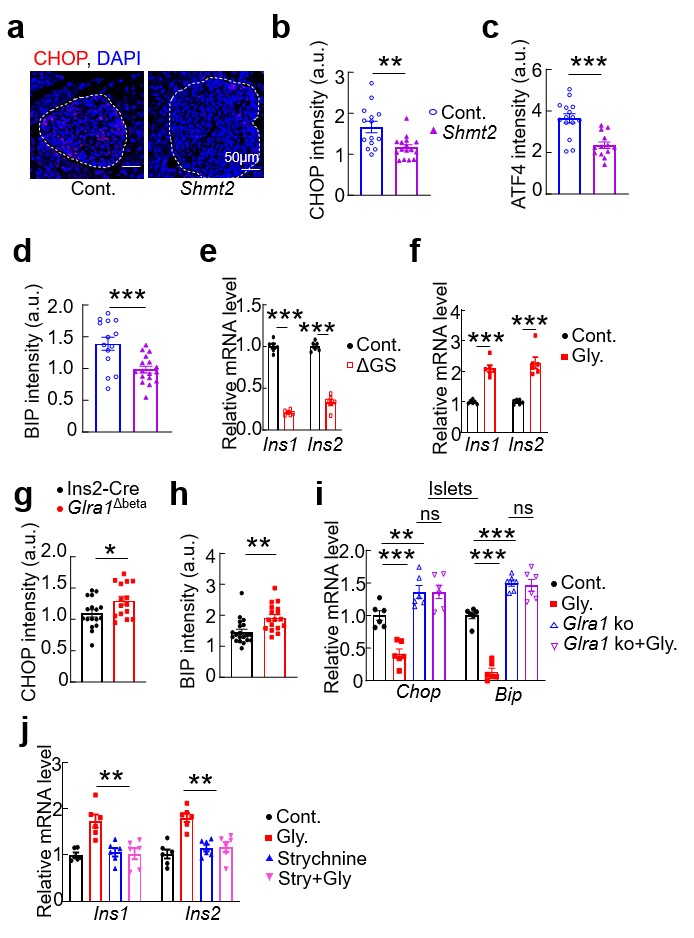


**Supplementary Figure S7** Glycine attenuates ER stress via GLRA1, related to Figure 3.

(a and b) Representative immunofluorescence images (a) and its quantification (b) of CHOP staining in pancreatic sections from HFD-fed mice overexpressing *Shmt2*. *n* = 14 and 16, respectively. Scale bar: 50 μm. (c and d) Quantification of ATF4 (c) and BIP (d) staining in pancreatic sections from HFD-fed mice overexpressing *Shmt2*. *n* = 15 and 13, respectively, in (c); *n* = 14 and 17, respectively, in (d). (e and f) Insulin mRNA levels under the conditions of glycine deficiency (e) or proficiency (f). *n* = 6 in all groups. (g and h) Quantification of CHOP (g) and BIP (h) staining in pancreatic sections from HFD-fed mice expressing or lacking *Glra1* in β-cells. *n* = 17 and 15, respectively, in (g). *n* = 20 and 17, respectively, in (h). (i) *Chop* and *Bip* mRNA levels in islets from HFD-fed mice expressing or lacking *Glra1* in β-cells, administered with vehicle or glycine. *n* = 6 in all groups. (j) Insulin mRNA levels following the glycine treatment in the presence or absence of strychnine (3 μmol/L). *n* = 6 in all groups. Data are expressed as mean ± SEM. ^*^*P* < 0.05; ^**^*P* < 0.01; ^***^*P* < 0.001; ns, not significant.


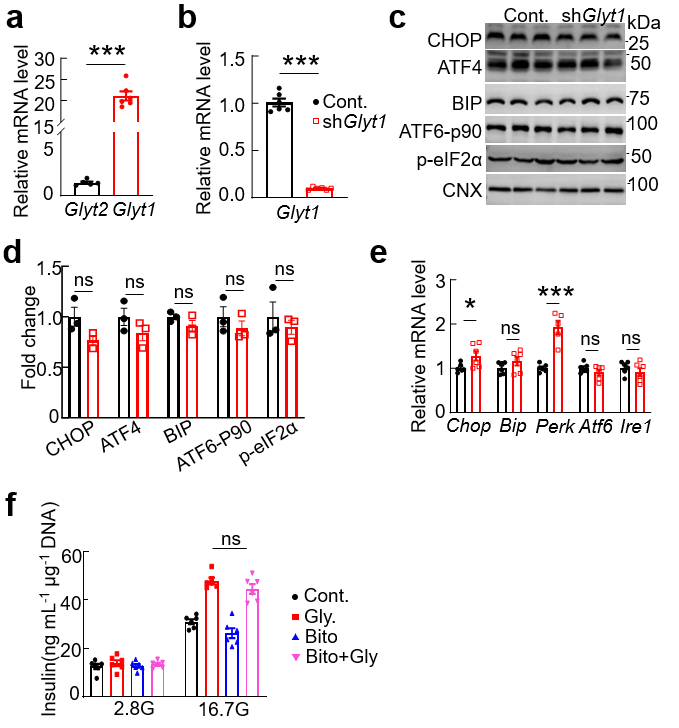


**Supplementary Figure S8** GlyT1 does not influence ER stress, related to Figure 4. (a) Relative glycine transporter mRNA levels in INS2 cells. *n* = 6 in both groups. (b) qPCR quantification of *Glyt1* knockdown efficacy. *n* = 6 in both groups. (c and d) Representative immunoblotting images (c) and quantification (d) of ER stress markers in INS1 cells in the presence or absence of *Glyt1*. The experiment was independently repeated for three times. (e) The mRNA levels of ER stress markers under *Glyt1* deficiency. *n* = 6 in both groups. (f) GSIS in INS1 cells in the presence of glycine transporter inhibitor, bitopertin (Bito, 100 μmol/L). *n* = 6 in all groups. Data are expressed as mean ± SEM. ^*^*P* < 0.05; ^**^*P* < 0.01; ^***^*P* < 0.001; ns, not significant.


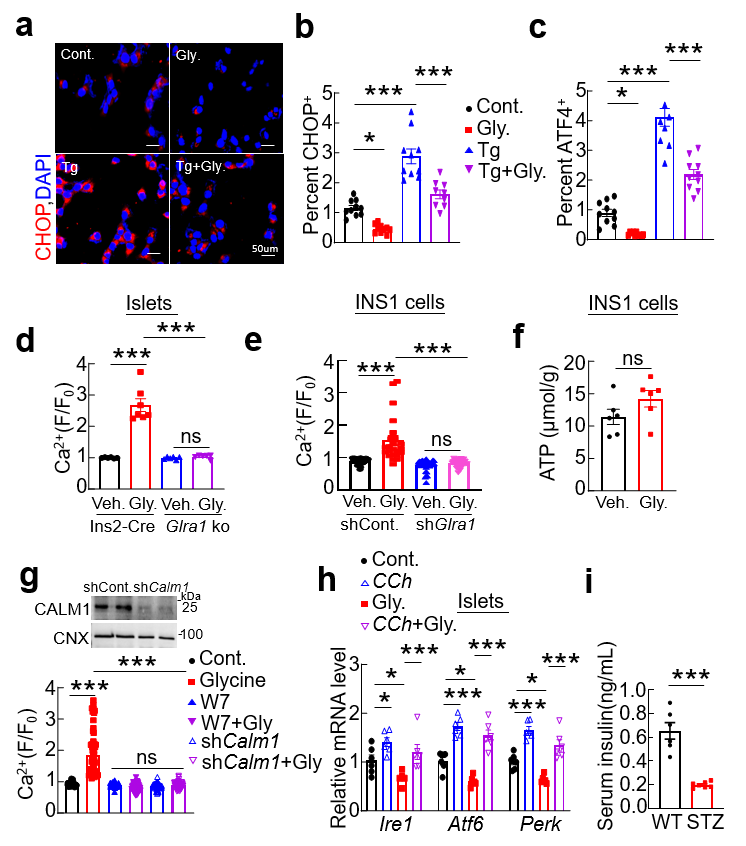


**Supplementary Figure S9** Glycine attenuates TG-induced ER stress, while IP_3_R activation abolishes glycine-mediated ER stress protection, related to Figure 5. (a and b) Immunofluorescence staining of ER stress marker. CHOP staining (a) and quantification (b) were done in the presence or absence of thapsigargin (Tg, 1 μmol/L) stimulation for 1 h, following by overnight treatment of 10 mmol/L glycine in INS1 cells. *n* = 10, 10, 10, and 9, respectively. Scale bar: 50 μm. (c) Quantification of ATF4 staining following co-administration of Tg and glycine. *n* = 10 in all groups. (d) Quantification of ER calcium flux in islets isolated from HFD-fed mice expressing or lacking *Glra1* in β-cells stained with Mag-Fluo-4 AM (20 μmol/L). 10 islets per well. *n* = 6, 7, 6, and 6, respectively. (e) Cytosolic calcium determined using Fluo-4AM in glycine-treated INS1 cells in the presence or absence of sh*Glra1*. *n* = 40, 23, 40, and 40, respectively. (f) ATP levels in INS1 cells treated with glycine for 24 h. *n* = 6 for both groups. (g) ER calcium in cells treated with calmodulin inhibitor W7 (50 μmol/L) or shRNA targeted against calmodulin. *n* = 65, 59, 62, 62, 61, and 65, respectively. (h) The mRNA levels of *Ire1, Atf6,* and *Perk* in islets from HFD-fed WT mice treated with glycine, following stimulation with CCh (1 mmol/L) to activate IP_3_R-mediated calcium release. *n* = 6 in all groups. (i) Fasting serum insulin levels in mice treated with or without STZ. *n* = 6 and 7, respectively. Data are expressed as mean ± SEM. ^*^*P* < 0.05; ^**^*P* < 0.01; ^***^*P* < 0.001; ns, not significant.


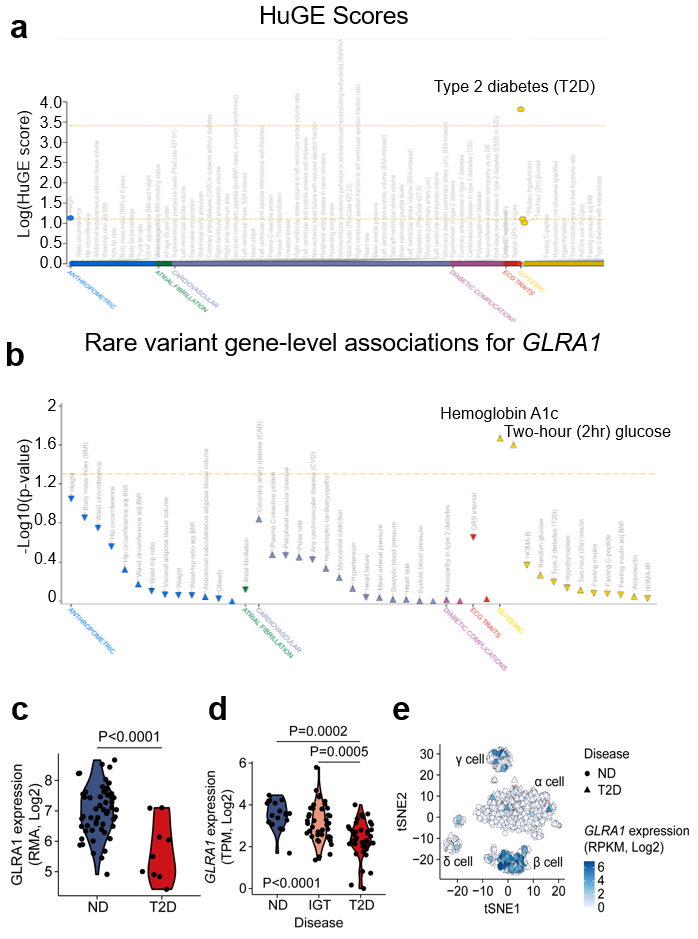


**Supplementary Figure S10** GLRA1 variants and expression associated with T2D in human, related to Figure 6. (a) HuGE scores of GLRA1 variants in T2D. (b) Rare variant gene-level associations for GLRA1. (c and d) GLRA1 expression in human islets from the public bulk transcriptomic datasets GSE38642 (c, ND: *n* = 54; T2D: *n* = 9), and GSE164416 (d, ND: *n* = 18; IGT: *n* = 41; T2D: *n* = 39). ND: non-diabetes; IGT: impaired glucose tolerance; T2D: type 2 diabetes. RMA: robust multiarray average; TPM: transcript per million. (e) Two-dimensional *t*-distributed stochastic neighbor embedding (*t*-SNE) projection of the endocrine cells (*n* = 1554) based on the expression values of the 500 genes with the highest biological variation across the endocrine cells from the single-cell RNA-seq dataset (E-MTAB-5061). The colour and shape correspond to *Glra1* expression (reads per kilobase of transcript per million mapped reads [RPKM], log-transformed) and disease (ND: *n* = 6; T2D: *n* = 4), respectively. Data are expressed as mean ± SEM. ns, not significant.

**Supplementary Table S1** Composition of amino acid-based high-fat diets (60% fat).

| **Formula** | **D12492 (Cont. HFD)** | **Glycine/serine free (ΔG/S HFD)** |
| --- | --- | --- |
| Kcal/g | 5.2 | 5.2 |
| Protein (% kcal from) | 18 | 17 |
| CHO (% kcal from) | 21 | 22 |
| Fat (% kcal from) | 61 | 61 |
| L-Cystine (g/kg) | 5.6 | 5.6 |
| L-Isoleucine (g/kg) | 9.9 | 9.9 |
| L-Leucine | 20.8 | 20.8 |
| L-Lysine (g/kg) | 17.2 | 17.2 |
| L-Methionine (g/kg) | 6.6 | 6.6 |
| L-Phenylalanine (g/kg) | 11.0 | 11.0 |
| L-Threonine (g/kg) | 9.4 | 9.4 |
| L-Tryptophan (g/kg) | 2.8 | 2.8 |
| L-Valine (g/kg) | 12.2 | 12.2 |
| L-Histidine-HCl-H_2_O (g/kg) | 6.0 | 6.0 |
| L-Alanine (g/kg) | 6.6 | 6.6 |
| L-Arginine (g/kg) | 7.8 | 7.8 |
| L-Asparagine-H_2_O (g/kg) | 9.3 | 9.3 |
| L-Aspartic Acid (g/kg) | 6.6 | 6.6 |
| L-Glutamine (g/kg) | 22.5 | 22.5 |
| L-Glutamic Acid (g/kg) | 27.4 | 27.4 |
| **Glycine (g/kg)** | **4.0** | **0.0** |
| L-Proline (g/kg) | 23.3 | 23.3 |
| **L-Serine (g/kg)** | **13.1** | **0.0** |
| L-Tyrosine (g/kg) | 11.9 | 11.9 |
| Corn starch (g/kg) | 0.0 | 17.1 |
| Maltodextrin 10 (g/kg) | 165.6 | 165.6 |
| Sucrose (g/kg) | 91.1 | 91.1 |
| Cellulose, BW200 (g/kg) | 66.2 | 66.2 |
| Soybean oil (g/kg) | 33.1 | 33.1 |
| Lard (g/kg) | 324.5 | 324.5 |

**Supplementary Table S2** List of the primers used in this study.

| **Primer** | **Sequence (5’→3’)** |
| --- | --- |
| Rat *Gapdh* | F- AAGGCCGGGGCCCACTTGAA |
|  | R- GGACTGTGGTCATGAGCCCTTCCA |
| Rat β-*actin* | F- GGAGATTACTGCCCTGGCTCCTA |
|  | R- GACTCATCGTACTCCTGCTTGCTG |
| Rat *Glra1* | F-ATCTCTGTCAAGGGTGCCAA |
|  | R-TCCCAGAGCCTTCACTTGTT |
| Rat *Glra2* | F-CCTGGGACCTTCTTTGCCTA |
|  | R- GCAGTTTTCTTCCTTGCCCA |
| Rat *Glra3* | F-TTGACTGCTGTTGTGCTTCC |
|  | R-GCTACAAACGTGCGAGTGAT |
| Rat *Glra4* | F-CAACAGTTTTGGCTCCGTCA |
|  | R-CCCATTCTTGAAGATGCGCA |
| Rat *Glrb* | F-GCTGATTGTGGTCCTCTCCT |
|  | R-GGACAACAGCATACTCCACG |
| Rat *Glyt1* | F-TGGAGGCTGTATGTGCTGAA |
|  | R-CATAGGGAAATGTGGCCGTG |
| Rat *Glyt2* | F-CCTATGCTGCCTCCTATGCT |
|  | R-CCAGTTAGGGTAGCGGTAGG |
| Rat *Ins1* | F-CCTGCTCGTCCTCTGGGAGCCCAAG |
|  | R- CTCCAGTGCCAAGGTCTGAAGATCC |
| Rat *Ins2* | F- CCTGCTCATCCTCTGGGAGCCCCGC |
|  | R- CTCCAGTGCCAAGGTCTGAAGGTCA |
| Rat *Chop* | F-ACGGAAACAGAGTGGTCAGT |
|  | R- AGACAGACAGGAGGTGATGC |
| Rat *Bip* | F-GAACCAACTCACGTCCAACC |
|  | R- CTTTCCCAAATACGCCTCGG |
| Rat *Perk* | F-TCTGTTCTGCCTTGGGATGT |
|  | R-CCGAAGTTCAAAGTGGCCAA |
| Rat *Atf6* | F-AGAGAAGCCTGTCACTGGTC |
|  | R-TAATCGACTGCTGCTTTGCC |
| Rat *Ire1* | F-CGGGAGAGCTGTGGTTAAGA |
|  | R-TCGGTAGGTGTGAGAGAGGA |
| Mouse *Actb* | F-GGAGATTACTGCCCTGGCTCCTA |
|  | R-GACTCATCGTACTCCTGCTTGCTG |
| Mouse *Pepck* | F-CAGGAAGTGAGGAAGTTTGTGG |
|  | R-ATGACACCCTCCTCCTGCAT |
| Mouse *Pc* | F-AGATGCACTTCCATCCCAAG |
|  | R-CCTTGGTCACGTGAACCTTT |
| Mouse *Pklr* | F-TCAAGGCAGGGATGAACATTG |
|  | R-CACGGGTCTGTAGCTGAGTG |
| Mouse *Hk1* | F-CGGAATGGGGAGCCTTTGG |
|  | R-GCCTTCCTTATCCGTTTCAATGG |
| Mouse *Gapdh* | F-AAGGCCGGGGCCCACTTGAA |
|  | R-GGACTGTGGTCATGAGCCCTTCCA |
